# Supplementary material for: Generation Healthy Kids: Protocol for a cluster-randomized controlled trial of a multi-component and multi-setting intervention to promote healthy weight and wellbeing in 6–11-year-old children in Denmark
Source: PLoS One. 2024 Dec 5;19(12):e0308142. doi: 10.1371/journal.pone.0308142 (PMC11620443; doi:10.1371/journal.pone.0308142)
Supplement: S2 File — (PDF) [file pone.0308142.s002.pdf]

SUPPORTING INFORMATION FILE 2

This file provides additional supporting information for the manuscript:
Thomsen et al. 2024: Generation Healthy Kids: Protocol for a cluster-randomized controlled trial of a multi-component and multi-setting intervention to promote healthy weight and wellbeing in 6–11-year-old children in Denmark

Table of contents

1. Municipalities included in each school invitation round ... 2
2. Requirements for school participation in Generation Healthy Kids ... 3
3. Details of Generation Healthy Kids intervention components ... 4
4. Description of measurement procedures ... 7
  4.1. Measurements at school ... 7
    4.1.1. Body composition and weight ... 7
    4.1.2. Height and waist circumference ... 7
    4.1.3. Blood pressure and resting heart rate ... 7
    4.1.4. Physical fitness and motor functions ... 7
    4.1.5. Cognitive functions ... 9
    4.1.6. Video-assisted child questionnaires ... 9
    4.1.7. School performance ... 10
    4.1.8. Blood samples ... 10
  4.2. Measurements at home ... 11
    4.2.1. Parental questionnaire ... 11
    4.2.2. MyFood24 dietary record ... 12
    4.2.3. Accelerometry ... 12
    4.2.4. Ethica application ... 13
5. References ... 14

1. MUNICIPALITIES INCLUDED IN EACH SCHOOL INVITATION ROUND

Supplementary Table 1 provides an overview of the municipalities from which we invited schools for the Generation Healthy Kids study.

Supplementary Table 1. List of municipalities included in each school invitation round for Generation Healthy Kids

Round	Region	Schools from the following municipalities invited
Round 1	Southern Denmark	Assens, Faaborg-Midtfyn, Nordfyns, Nyborg and Odense.
	Capital Region	Ballerup, Egedal, Fredensborg, Gladsaxe, Hillerød, Hvidovre, Ishøj, København, Rødovre and Tårnby.
Round 2	Southern Denmark	Fredericia, Kerteminde, Middelfart, Kolding and Vejle.
	Capital Region	Brøndby, Frederiksberg, Halsnæs, Lyngby-Tårbæk, Rudersdal and Vallensbæk.
Round 3	Southern Denmark[a]	Billund, Haderslev, Langeland, Tønder, Varde, Vejen and Aabenraa.
	Capital Region[b]	Allerød, Dragør, Furesø, Gentofte, Glostrup, Gribskov, Helsingør, Herlev, Høje-Taastrup and Hørsholm.
	Zealand Region[c]	Greve, Holbæk, Køge, Lejre, Ringsted, Roskilde, Slagelse, Solrød and Sorø.

[a]All remaining municipalities from Region of Southern Denmark invited, except Svendborg who was excluded due to existing school-based child health promotion project, and Fanø, Ærø, Esbjerg and Sønderborg who were excluded due to far travel distance from the study centers.

[b]All remaining municipalities from the Capital Region invited, except Albertslund and Frederikssund who were excluded due to an existing school-based child health promotion project, and Bornholm who was excluded due to far travel distance from the study centers.

[c]Nine selected municipalities from Zealand Region invited based on closest geographical proximity to study centers.

2. REQUIREMENTS FOR SCHOOL PARTICIPATION IN GENERATION HEALTHY KIDS

Supplementary Table 2 shows an overview of the requirements for schools to participate in Generation Healthy Kids.

Supplementary Table 2. List of requirements for schools to participate in Generation Healthy Kids

Requirement	Which schools?
**Project coordination, participant recruitment and data collection**<br>• School management and relevant staff must participate in coordinating meetings and help communicate about the project to parents throughout the project period.<br>• The school must deliver names and personal identification numbers of children in the included classes to the research group before project start.<br>• Teachers should participate in information meetings for families held at the school before project start.<br>• The school must allocate student time and provide rooms for measurements during school hours three times during the project period.<br>• The school must deliver data on child absence during the project period (only children whose parents provide informed consent).<br>• School management and relevant staff must participate in evaluation activities regarding the project (questionnaires and interviews).	Intervention and control
**Courses**<br>• Teachers and pedagogues must allocate time for participation in courses on diet and meal practices (4 hours), physical activity (6 hours) and screen media and sleep (3 hours).	Intervention only
**School lunch**<br>The school must support the implementation of school lunch four days per week, which includes:<br>• The school must allocate 40–45 minutes for lunch break.<br>• Class teachers and pedagogues must help facilitate the lunch, i.e., follow food pedagogical principles and act as "meal hosts".<br>• The school must hire a part-time person 15-25 hours per week (depending on school size) to support the lunch program, including receiving the food, simple preparation, arranging food on trolleys, washing-up, and performing hygienic self-checks. This expense can be partly covered by the project.<br>• Existing kitchen facilities must be of a sufficient standard to ensure that the lunch program is feasible within the project's budget.	Intervention only
**Other intervention elements**<br>• The school must implement 3x40 minutes of weekly physical activity sessions according to FIT FIRST 10 concept during the entire project period.<br>• The school must implement teaching sessions developed and provided by GHK on food literacy and screen media habits in the classroom.<br>• The school must provide facilities/rooms for 4–6 parent workshops or -events after school hours during the project period.	Intervention only

3. DETAILS OF GENERATION HEALTHY KIDS INTERVENTION COMPONENTS

Supplementary Table 3 provides a description of the Generation Healthy Kids intervention components, including setting (school, afterschool clubs, family, and/or local community), focus area (diet, physical activity, screen media use or sleep), type (core or co-created component), target groups, timing, and frequency of each component.

Supplementary Table 3. Description of Generation Healthy Kids intervention components

Setting	Component type[a]	Focus of intervention component[b]	Description of intervention component	Responsible for delivering intervention component	Target group for intervention component	Timing and frequency of intervention component
**School**	**Core**	Diet	Free-of-charge school lunch	External supplier, teachers and pedagogues	Children	Four days per week during school year 1 and 2.[c]
			Course for school staff on principles and practical aspects of school lunch program	Research group	Teachers and pedagogues	4-hour course at start of school year 1. Catch-up course for new teachers at start of school year 2.
			Classroom exercises on food literacy	Teachers	Children	Materials delivered to schools once at beginning of school year 1. Frequency of use decided by teachers.
		Physical activity	3x40 minutes per week of vigorous physical activity during school hours (FITFIRST10)	Teachers and pedagogues	Children	Three 40-minute sessions per week during school year 1 and 2.
			Course for school staff on FITFIRST10	Research group	Teachers and pedagogues	6-hour course at start of school year 1. Online catch-up courses available, and catch-up courses arranged on request from schools.
			Packages of loose play and sports equipment for use in recess distributed to classes.	Teachers and pedagogues	Children	Packages delivered once at the beginning of school year 1. Frequency of use decided by school staff.
		Screen media use and sleep	Classroom exercises on screen media use and sleep.	Teachers	Children	Three teaching sessions three times during intervention period (once in school year 1, twice in school year 2).
			Course for school staff on children's screen media habits and sleep.	Research group	Teachers	3-hour course at start of school year 1.

Suppl. Table 3 (continued)

Setting	Component type[a]	Focus of intervention component[b]	Description of intervention component	Responsible for delivering intervention component	Target group for intervention component	Timing and frequency of intervention component
**School**	Co-created	Diet, physical activity, screen media use, sleep	Distribute an inclusive guide designed to support the development of school policies within GHK focus areas: diet, physical activity, screen media use, and sleep.	Research group	School management, school board	Initiated in school year 2. Frequency depends on local context.
		Physical activity	Education of older pupils as "play patrols" during recess.	Research group	Children	Initiated in school year 2. Frequency depends on local context
**After-school clubs**	Co-created	Physical activity	Improved structures for active outdoor play in afterschool clubs, e.g. lines for ball games and small goals for football.	Research group	Children	Initiated in school year 2. Frequency depends on local context.
		Diet	Dialogue and inspiration to improve meal practices and nutritional quality of breakfast and afternoon meals in afterschool clubs	Research group	Afterschool club management and staff	Initiated in school year 2. Frequency depends on local context.
			Activities in afterschool clubs focusing on healthy foods and improved food competence, e.g. excursions, quizzes, and afternoon events.	Research group After-school club staff	Children	Initiated in school year 1. Frequency depends on local context.
**After-school clubs and local community**	Co-created	Physical activity	Summer camps incorporating physical activity, focusing on children with limited sporting experience, organized in collaboration with afterschool clubs and local sports organizations.	Research group, local sports organizations, afterschool club staff	Children	Summer holiday between school year 1 and 2. One week at each intervention school.
			Collaborations with sports clubs to increase access to leisure time sports for children not currently members, e.g. advertising existing free pass schemes and organizing visits from local sports clubs in afterschool clubs.	Research group, local sports organizations, afterschool club staff	Children Parents	Initiated in school year 2. Frequency depends on local context.
**School and local community[d]**	Co-created	Overall child health and well-being	"Future Workshops" with children in school to gather children's own perspectives on barriers and facilitators for thriving and living a "healthy" life and suggestions for local action.	Research group	Children	Initiated in school year 2. Action ideas delivered to school leader and local community groups mid-school year 2.
**Family**	Core	Screen media use and sleep	Written inspirational and educational material on screen media use and sleep distributed electronically.	Research group	Parents	Materials distributed once in school year 1 and twice in school year 2.
		Diet	Written inspirational and educational materials on healthy eating and snacking habits distributed electronically.	Research group	Parents	Materials distributed once in school year 1.

Suppl. Table 3 (continued)

Setting	Component type[a]	Focus of intervention component[b]	Description of intervention component	Responsible for delivering intervention component	Target group for intervention component	Timing and frequency of intervention component
**Family**	Core	Diet, physical activity, screen media use, sleep	Family event for parents and children focusing on diet, physical activity, screen media habits and sleep	Research group	Children<br>Parents	Organized once at each intervention school during school year 1.
		Screen media use and sleep	Parent workshops on children's screen media use and sleep practices	Research group	Parents	One workshop in school year 1, two workshops in school year 2.
	Co-created	Diet	Family cooking workshops where parents and children cook simple, healthy meals together.	Research group	Children<br>Parents	Depends on local context, needs and relevance.
**Local community[d]**	Co-created	Overall child health and well-being	Stakeholder analysis and mapping of local stakeholders.<br>Two Group Model Building (GMB)-workshops using STICKE.<br>Establishment of local community partnership groups to develop and implement local action ideas.	Researchers deliver workshops.<br><br>Local stakeholders design and implement local action ideas, supported by research team.	Local stakeholders with influence on child health and wellbeing[e]	GMB workshops and establishment of local community partnership groups in school year 1.<br><br>Implementation of local action ideas in school year 1 and 2.
		Diet	Nudging, pricing and product placement interventions to promote sales of healthy foods in local supermarkets.	Local supermarkets, supported by research group	Customers	Initiated in school year 1. Frequency depends on local context.
		Diet	Decrease portion sizes and increase availability of healthy alternatives in local restaurants, cafeterias and fast-food outlets	Local restaurants and fast-food outlets, supported by research team.	Customers	Initiated in school year 2. Frequency depends on local context.

[a]The "core" intervention elements will be predefined based on pre-existing evidence and experience from the GHK pilot study. These intervention elements will be implemented uniformly at all intervention schools, and they are mandatory for the research group and school staff to deliver (see Additional File 2, Suppl. Table 2 for an overview of requirements for school participation). "Co-created" elements will be developed in collaboration with each intervention school and community. These elements may thus differ between schools and communities depending on local needs, relevance, and resources.

[b]The focus areas of GHK are diet, physical activity, screen media use and sleep. Some intervention components target only one focus area, while others target several focus areas simultaneously.

[c]For logistic reasons, the school lunch program will start successively at intervention schools. Six schools will start in Month 1-2 (Oct-Nov 2023), and six schools will start in Month 4 (Jan 2024) of the intervention period. This will allow sufficient time for close implementation support from the research team at the beginning of the school lunch program.

[d]The local community intervention will take place in the local community around eight of the 12 intervention schools.

[e]Local stakeholders invited for workshops will include municipality representatives, school leader, school board, parent representatives, leisure time organizations, sports clubs, social housing organizations, other NGOs, and food retailers.

Abbreviations: GHK: Generation Healthy Kids; GMB, Group Model Building; STICKE: Systems Thinking in Community Knowledge Exchange.

4. DESCRIPTION OF MEASUREMENT PROCEDURES

The following sections provide a detailed description of measurement procedures used in the study.

4.1. Measurements at school

4.1.1. Body composition and weight

Body composition (i.e., fat mass and fat free mass) will be measured by air-displacement plethysmography using a BODPOD and a bioimpedance analyzer (InBody 270). The latter will also be used to measure body weight. The children will be wearing swimwear/underwear (or alternatively light clothes) and a bathing cap in the BODPOD.

4.1.2. Height and waist circumference

Standing height and waist circumference will be measured in triplicate to the nearest millimetre using a portable stadiometer and a non-elastic measuring tape at the level of the umbilicus, respectively. The mean of the three measurements will be used.

4.1.3. Blood pressure and resting heart rate

Resting blood pressure and heart rate will be measured three times by an automated device in the supine position after 10 minutes of rest. The appropriate cuff size for the child's arm circumference will be used. The mean of the three measurements will be used.

4.1.4. Physical fitness and motor functions

Aerobic fitness

Children's cardiovascular fitness will be evaluated by the Yo-Yo Intermittent Recovery Level 1 Children's test (Yo-Yo IR1C) (1), where the children will have to run 2x16 m at increasing speed with a 10-s break between each shuttle run. The test continues until the children cannot reach the finish line in time and takes around 20 min. for up to 50 pupils, depending on the size of the indoor gym. Total running distance (m) will be recorded as a measure of cardiovascular fitness. Heart rate will be measured continuously during the test to determine maximal heart rate. The test is simple and validated for fitness and maximal heart rate assessment for 6-10-year-old children (1).

Muscular strength, jump height and sprint performance

Muscle strength, jump height and sprint performance will be determined by a handgrip strength test, a countermovement jump test and a 20-m sprint test. The hand grip strength test is conducted with a hand grip dynamometer. The child squeezes the dynamometer with all their strength, two times with each hand. An average score is then calculated using the measurements from both hands. Jump height is assessed based on maximal countermovement jump performance. The child stands on a force plate and places both hands at the waist. From this position, the child jumps as high as possible by performing a vertical countermovement jump. The jump height is calculated based on takeoff-velocity calculated from the vertical force (Fz). Sprint time is measured by photocells. The children will complete each test twice and will be instructed to perform their best.

Postural control and gross motor functions

Balance ability is measured as summed sway path during 30 s maintained quite stance on a force plate. Lower extremity gross motor function is tested by use of the Y-balance task in which reaching distance is tested for both right and left foot during unilateral stance on the contralateral foot. Two attempts are allowed for each trial anterior and posterior-lateral directions, and the sum of reaching distance is quantified as the test result.

Upper extremity gross-motor function is tested as speed and accuracy in the ability to perform accurate, goal-directed bilateral arm reaching movements towards visually displayed targets. The sum of correct buttons pressed during 30 s is quantified and two attempts performed for each participating child.

Agility and motor skill assessment

Ability to combine motor skills is measured as criterion-referenced skill performance and completion time to complete the Canadian Agility and Movement Skill Assessment (CAMSA) test (2) with different motor tasks incorporated. The test is known as the Canadian Agility and Movement Skill Assessment (CAMSA). The test has been validated in Danish schoolchildren and found age-appropriate (3).

4.1.5. Cognitive functions

The children will complete a short battery of standardized, validated and age-appropriate neurocognitive tests including tests of processing speed, executive functions, memory and sustained attention. The tests are all based on the CANTAB battery (Cambridge Cognition, UK). All tests are administered individually in a Danish version via iPad (4).

4.1.6. Video-assisted child questionnaires

Due to the respondents' low age and ability to read, a video- and speech assisted questionnaire will be administered. The children will fill in the questionnaire using tablets and headphones enabling all items of the questionnaire to be read aloud, while the text appears on the screen, and the introduction is supported by photos. The response options will be illustrated with smileys or illustrations.

<u>KIDSCREEN-27</u>

Children's well-being will be measured using KIDSCREEN-27. This validated questionnaire consists of 27 items that measure five dimensions of children's quality of life and well-being. These are physical wellbeing (5 items), psychological wellbeing (7 items), autonomy and parent relations (7 items), relations to peers and social support (4 items), and quality of life in the school environment (4 items) (5).

<u>MyPL (My Physical Literacy) and leisure sport participation</u>

A Danish video-assisted questionnaire, the MyPL questionnaire, validated for children down to 7 years of age will be used to measure children's intrinsic motivation and confidence for physical activity as well as their knowledge and understanding of physical activity. The MyPL is a 20-item physical literacy assessment tool that strives to account for how the domains of physical literacy (motivation, confidence, physical competence, and knowledge in relation to physical activity) differs across different social and physical environments for physical activity, as described in the conceptualization of physical literacy by Whitehead. This structure makes it cognitively easier for the children to think about and thus answer. The questionnaire consists of five subscales reflecting context and discipline-specific motivation, confidence, and knowledge about physical activity. The children will also be asked about their participation in leisure time sports club activities.

Food literacy

Children's food literacy and perceived school and family meal culture will be assessed using a self-constructed questionnaire developed for and validated in the age group. The questionnaire will measure three dimensions of children's food literacy 1) to do, 2) to sense, and 3) to know (6,7) and perceived school class meal culture (e.g., time to eat; activities while eating (use of tablets, books read aloud, etc.); and the teachers' participation in the lunch meal) and family meal culture (e.g. eating alone/together, and looking at screens while eating).

4.1.7. School performance

School performance will be assessed by use of standard and age-appropriate tests of mathematics proficiency and reading comprehension (4,8). Both of these tests are administered individually in Danish (Hogrefe, Denmark).

4.1.8. Blood samples

Collection of blood samples

Blood samples will be collected in the overnight fasted state (except water) only from children in the Capital and Zealand Regions whose legal guardians have provided informed consent for blood samples. The children can have breakfast shortly after the blood samples have been collected. The children will be provided with local anesthetic patches (EMLA), which the parents will be instructed to apply on their child's arms in the morning. After removal of the patches, max. 30 ml blood will be drawn from the child's forearm by trained staff. The blood samples will be brought back to University of Copenhagen for processing and storage at maximum –70° C until analysis.

Planned blood sample analyses

Supplementary Table 4 gives an overview of the planned analyses of the blood samples.

Supplementary Table 4. Planned blood sample analyses

Biomarker field	Analyses
**Diet & nutrition biomarkers**	• Fatty acids, lipids and other nutrient biomarkers<br>• Alkylresorcinols (wholegrains) and other food biomarkers<br>• 25-hydroxyvitamin D, vitamin D binding proteins and metabolites<br>• Haemoglobin, ferritin, and transferrin receptor (iron)
**Growth & development**	• IGF-I, IGFBP-3 and other growth factors<br>• Osteocalcin, bone specific alkaline phosphatase and other bone markers<br>• Parathyroid hormone<br>• Sex hormones
**Cardiometabolic**	• Triacylglycerol and total, LDL and HDL cholesterol and others<br>• Glucose, insulin, C-peptide, glycosylated hemoglobin (HbA1c) etc.
**Appetite**	• Appetite hormones
**Inflammation**	• C-reactive protein, cytokines, adipokines, and related markers<br>• Immune cells and immune markers
**Cognition**	• Blood markers related to brain function such as brain-derived neurotrophic factor (BDNF) and serotonin
**Mechanisms**	• Metabolomics, proteomics and lipidomics<br>• Short chain fatty acids and other metabolites
**Genetics**	• Genotypes and epigenetics

4.2. Measurements at home

4.2.1. Parental questionnaire

Supplementary Table 5 provides an overview of the items in the parental questionnaire at each measurement round.

Supplementary Table 5. Overview of items in parental questionnaire at each measurement round

	**Round 1***	**Round 2***	**Round 3***
Child food allergies (intervention schools only), bandage allergies and chronic diseases	X		
Child's neurodevelopmental disorders and medical conditions	X		
Family sociodemographic background	X		
Parents' height and weight	X		
Child's birth weight, length and gestational age	X		
Child's pubertal stage	X		X
Child Strength and Difficulties questionnaire	X	X	X
Child's leisure time sports participation	X	X	X
Child's breakfast consumption, beverages, fish intake and dietary supplements	X	X	X
Modified SCREENS questionnaire	X	X	X

Children's Sleep Habits Questionnaire	X	X	X
Family grocery shopping habits	X		X
Parent's perception of the project and activities		X	X
Adverse events and concomitant medicine		X	X

*Round 1: Sep–Nov. 2023; Round 2: May–June 2024; Round 3: May–June 2025.

Child Strength and Difficulties Questionnaire

Children's psychological wellbeing will be measured using the parent-reported version of the Strengths and Difficulties Questionnaire (9). This parental questionnaire will be used to supplement child-reported quality of life and wellbeing (which is assessed by KIDSCREEN-27 as described above). An overall total difficulties score is calculated, along with five subscale scores: emotional symptoms, conduct problems, hyperactivity/inattention, relationship problems, and prosocial behavior.

Children's Sleep Habits Questionnaire

Children's sleep behaviors will be assessed using the Danish translation of the Children's Sleep Habits Questionnaire (10). These data will supplement the accelerometry data (Axivity AX3 accelerometer) in the assessment of children's sleep behaviors.

Modified SCREENs questionnaire

Children's and their parent's screen time, type and current screen time practices and behaviors will be collected using a modified version of the parent-reported SCREENS questionnaire (11).

4.2.2. MyFood24 dietary record

Children's daily intake of food and drinks will be recorded by the parents for three consecutive days (two weekdays and one weekend day). We will use the validated, web-based tool, myfood24®, which uses data from national food composition tables. The tool is user-friendly, suitable for the target group and recording links can be sent via email.

4.2.3. Accelerometry

Detailed, objective information on the whole spectrum of behaviors will be assessed by continuous 24-hour/7-8-day Axivity® accelerometer measurement using thigh positioned monitors. Using the thigh measurement position, the modified age-adjusted method of Skotte et al. (12) provides the ability to classify postural allocations like sitting or standing and activities like walking and running

with high sensitivity and specificity in children (13). Time lying in bed and sleep time will be objectively measured using an accelerometer classification algorithm currently under development.

The Axivity® AX3 monitor is a small (23 x 32.5 x 7.6 mm), lightweight (11 g), waterproof, 3-axis accelerometer data logger (Axivity, 2016). The Axivity® AX3 will be attached to the skin using thigh belts developed by a Danish company (Elas) or tape (Opsite Flexifix®, Fixumull®) directly on the body using compress (gaze) or artificial skin (DuoDerm) between the monitor and the skin. Children with plaster allergy, childhood eczema or sensitive skin will be offered to use belts. The children or parents will receive an information leaflet with relevant information regarding the physical activity measurement.

4.2.4. Ethica application

At baseline, we ask parents to install a research-based app, Ethica (https://ethicadata.com), on the participating children's own smartphones and/or tablets. At all test rounds, we will use Ethica to collect information on screen time on a second-to-second basis over a period of 14 days. After installation, the app will run in the background on the device, and data will be sent encrypted to a secure server. The app has been developed to meet legal requirements for data security.

5. REFERENCES

1. Bendiksen M, Ahler T, Clausen H, Wedderkopp N, Krustrup P. The use of yo-yo intermittent recovery level 1 and andersen testing for fitness and maximal heart rate assessments of 6-to 10-year-old school children. J Strength Cond Res. 2013;27(6):1583–90.

2. Longmuir PE, Boyer C, Lloyd M, Borghese MM, Knight E, Saunders TJ, et al. Canadian Agility and Movement Skill Assessment (CAMSA): Validity, objectivity, and reliability evidence for children 8-12 years of age. J Sport Heal Sci. 2017;6:231–40.

3. Elsborg P, Melby PS, Kurtzhals M, Tremblay MS, Nielsen G, Bentsen P. Translation and validation of the Canadian assessment of physical literacy-2 in a Danish sample. BMC Public Health. 2021;21(1):1–9.

4. Geertsen SS, Thomas R, Larsen MN, Dahn IM, Andersen JN, Krause-Jensen M, et al. Motor Skills and Exercise Capacity Are Associated with Objective Measures of Cognitive Functions and Academic Performance in Preadolescent Children. PLoS One. 2016; 11(8):e0161960.

5. Ravens-Sieberer U, Auquier P, Erhart M, Gosch A, Rajmil L, Bruil J, et al. The KIDSCREEN-27 quality of life measure for children and adolescents: Psychometric results from a cross-cultural survey in 13 European countries. Qual Life Res. 2007;16(8):1347–56.

6. Benn J. Food, nutrition or cooking literacy - a review of the concept and competencies regarding food education. Int J Home Econ. 2014;7(1):13–35.

7. Stjernqvist NW, Elsborg P, Ljungmann CK, Benn J, Bonde AH. Development and validation of a food literacy instrument for school children in a Danish context. Appetite. 2021;156:1–10.

8. Sorensen LB, Dyssegaard CB, Damsgaard CT, Petersen RA, Dalskov SM, Hjorth MF, et al. The effects of Nordic school meals on concentration and school performance in 8- to 11-year-old children in the OPUS School Meal Study: A cluster-randomised, controlled, cross-over trial. Br J Nutr. 2015;113(8):1280–91.

9. Goodman R. Psychometric properties of the strengths and difficulties questionnaire. J Am Acad Child Adolesc Psychiatry. 2001;40(11):1337–45.

10. Owens JA, Spirito A, McGuinn M. The Children's Sleep Habits Questionnaire (CSHQ): Psychometric properties of a survey instrument for school-aged children. Sleep. 2000;23(8):1043–51.

11. Klakk H, Wester CT, Olesen LG, Rasmussen MG, Kristensen PL, Pedersen J, et al. The development of a questionnaire to assess leisure time screen-based media use and its proximal correlates in children (SCREENS-Q). BMC Public Health. 2020;20(1):1–12.

12. Skotte J, Korshøj M, Kristiansen J, Hanisch C, Holtermann A. Detection of Physical Activity Types Using Triaxial Accelerometers. J Phys Act Heal. 2014;11(1):76–84.

13. Brønd JC, Grøntved A, Andersen LB, Arvidsson D, Olesen LG. Simple Method for the Objective Activity Type Assessment with Preschoolers, Children and Adolescents. Children. 2020;7:72.
