# Supplementary material for: Generation Healthy Kids: Protocol for a cluster-randomized controlled trial of a multi-component and multi-setting intervention to promote healthy weight and wellbeing in 6–11-year-old children in Denmark
Source: PLoS One. 2024 Dec 5;19(12):e0308142. doi: 10.1371/journal.pone.0308142 (PMC11620443; doi:10.1371/journal.pone.0308142)
Supplement: S3 File — (PDF) [file pone.0308142.s003.pdf]

Generation Healthy Kids

Main study

[Generation Sunde Børn - hovedstudie]

Version: 3.0

Date: 25-07-2023

1 Responsible parties

Principal Investigator:
Professor, Jens Troelsen
Head of Department of Sports Science and Clinical Biomechanics
Faculty of Health Sciences, University of Southern Denmark
Campusvej 55, 5230 Odense M, Denmark
Phone: + 45 65 50 34 93
E-mail: JTroelsen@health.sdu.dk

Co-PI
Professor, Nikolai Baastrup Nordsborg
Head of Department of Nutrition, Exercise and Sports
University of Copenhagen
Nørre Allé 51, 2200 København N, Denmark
Phone: + 45 35 32 16 12,
E-mail: nbn@nexs.ku.dk

Investigators:
Professor, Ulla Toft
Center for Clinical Research and Prevention
Bispebjerg and Frederiksberg Hospital
Nordre Fasanvej 57
2000 Frederiksberg, Denmark
Phone: +45 29 99 78 77
E-mail: Ulla.toft@regionh.dk

Associate Professor, Camilla Trab Damsgaard
Department of Nutrition Exercise and Sports
Faculty of Science, University of Copenhagen
Rolighedsvej 26, 1958 Frederiksberg C, Denmark
Phone: +4535332221
E-mail: ctd@nexs.ku.dk

Professor, Peter Krustrup
Department of Sports Science and Clinical Biomechanics
Faculty of Health Sciences, University of Southern Denmark
Campusvej 55, 5230 Odense M, Denmark
Phone: +45 21 16 15 30
E-mail: pkrustrup@health.sdu.dk

Professor, Anders Grøntved
Department of Sports Science and Clinical Biomechanics

Faculty of Health Sciences, University of Southern Denmark
Campusvej 55, 5230 Odense M, Denmark
Phone: +45 65 50 44 65
E-mail: agroentved@health.sdu.dk

Senior Research Fellow, Rikke Fredenslund Krølner
Centre for Intervention Research
University of Southern Denmark/National Institute of Public Health
Studiestræde 6, 1455 Copenhagen K
Phone: +45 65 50 78 41
E-mail: rikr@sdu.dk

GCP coordinator:
Le Lene Susanne Simon Stevner
Department of Nutrition, Exercise and Sports,
Faculty of Science, University of Copenhagen
Rolighedsvej 26, 1958 Frederiksberg C, Denmark
Phone: +45 3533 2371
E-mail: less@nexs.ku.dk

Medical Expert and Clinically Responsible Physician:
Professor, MD, Christian Mølgaard
Department of Nutrition, Exercise and Sports,
Faculty of Science, University of Copenhagen
Rolighedsvej 26, 1958 Frederiksberg C, Denmark
Phone: +45 35 33 25 16
E-mail: cm@nexs.ku.dk

2 Table of Contents

1 Responsible parties 2
2 Table of Contents 4
3 Abbreviations and Definition of Terms 7
4 Background 8
5 Study Objectives 12
5.1 Overall Objective 12
5.2 Specific Objectives 12
6 Investigational Plan 14
6.1 Study Design 14
6.1.1 Justification of study design 14
6.1.2 Randomization 14
6.1.3 Timeframe of the trial 15
6.1.4 Sample size justification 15
6.1.5 Blinding 16
6.2 Study Participants 16
6.2.1 Inclusion Criteria 16
6.2.2 Exclusion Criteria 17
6.2.3 Withdrawal Criteria 17
6.2.4 Recruitment of schools 18
6.2.5 Recruitment and enrolment of study participants 18
6.3 Intervention 22
6.3.1 Principles of the intervention 22

6.3.2 Interventions in schools and after-school clubs 24

6.3.3 Interventions focused on the child through the family 28

6.3.4 Intervention focused on the local environment 30

6.3.5 Compliance 31

6.4 Control schools 32

6.5 Overview of study procedures and activities 32

6.6 Measurements and study procedures 35

6.6.1 Questionnaires to be filled in at home 35

6.6.2 Other procedures at home 36

6.6.3 Measurements in school 37

6.6.4 Evaluation of measures related to implementation of the intervention and organizational aspects 40

6.6.5 Measurements at further school, community, and national level 41

6.6.6 Use of the school's recordings of children's absence 42

6.7 Adverse Events 42

6.8 Handling of biological samples 42

6.8.1 Sample analysis 42

6.8.2 Genotypes and epigenetic analyses 44

6.8.3 Research Biobank 44

6.8.4 Biobank for future research purposes 44

6.9 Data Management and Quality Control 45

6.10 Use of data from Danish registers 47

6.11 Statistical methods 48

6.12 Suspension or early termination of the study 49

6.13 Serious adverse events and reporting 50

6.13.1 Serious adverse events 50

6.13.2 Reporting of Serious adverse events 50

6.14 Study Documentation 50

6.14.1 Publication of Results 50

7 Ethical conduct of the study 51

7.1 Ethical approval and study registration 51

7.2 Justification of the study 51

7.3 Underage study participants - justification and precautions 51

7.4 Children of ethnic minority 52

7.5 Risks and safety in relation to conducting the study 52

7.6 Benefits of the study and risk-benefit evaluation 53

7.7 Participant Information and Informed Consent 54

7.8 Protocol Changes 54

7.9 Protocol Deviations 55

7.9 Insurance 55

8 Funding 55

9 Remuneration for study participants 55

10 References 57

11 List of Appendices (all in Danish) 68

3 Abbreviations and Definition of Terms

AE	Adverse Event
BMI	Body Mass Index
CCRP	Center for Clinical Research and Prevention
CRF	Case Report Form
DK-East	The Capital Region of Denmark and Region Zealand
DK-West	The Region of Southern Denmark
FFM	Fat free mass
FM	Fat mass
GDPR	General Data Protection Regulation
LCPG	Local Community Partnership Group
MVPA	Moderate to Vigorous Physical Activity
NEXS	Department of Nutrition, Exercise and Sports
PA	Physical Activity
PE	Physical Education
PL	Physical Literacy
SDQ	Strengths and Difficulties Questionnaire
SDU	University of Southern Denmark
SES	Socioeconomic Status
SFO	After School Club
UCPH	University of Copenhagen
VPA	Vigorous Physical Activity

4 Background

4.1 The global problem of overweight and obesity

Overweight and obesity among children is a global problem affecting more than 41 million children under the age of 5 years. The World Health Organization (WHO) describes childhood obesity as one of the most serious public health challenges worldwide for the 21st century (1,2). The prevalence of overweight and obesity in Danish children is 12-13% in primary school and increases to 18-19% at the time of graduation (3), why early prevention is important. In the adult population 51% are estimated to be overweight or obese (4,5).

Overweight and obesity have many negative health consequences for children during their early years and in later life. For instance, children who have overweight or obesity are more likely to experience bullying, stigmatization and suffer from low self-esteem, anxiety, and lower quality of life (6). In addition, children with overweight and obesity have an increased risk of overweight and obesity as adults, which is well known to lead to poorer health and well-being and marked economic negative effects for the individual and for society.

There are stark inequalities in the prevalence of childhood overweight, both worldwide and in Denmark, contributing to the health inequality found in most countries (4,7). In Denmark, the overweight prevalence among children aged 6-7 years in high-income families is 11% compared to 26% in low-income families. Among children aged 14-15 years, the prevalence of overweight is almost 30% in families with short education compared to 10% among those from families with long education (8).

Many trials have been conducted to reduce childhood overweight and obesity, and several comprehensive systematic reviews have investigated the effect of different interventions in preventing childhood overweight and obesity (9–16). However, most of the earlier studies find no or only small effects on children's body mass index (BMI), and the evidence of long-term effects is limited.

Thus, there is a strong need to develop new innovative approaches and programs that effectively can prevent overweight among all children, starting early in life and embedding promising programs and integrated approaches into everyday practices.

4.2 Important characteristics to increase likelihood of success

Some of the earlier studies did show a significant effect on overweight measures. Across these studies, some characteristics and elements seem to increase the likelihood of success:

- A multi-component, multi-setting intervention, including well-planned involvement of relevant stakeholders, is likely to yield the highest level of effect in promoting healthy weight in children (17,18).
- Multi-component interventions targeting two or more health behaviours (i.e., physical activity, dietary outcomes, sedentary behaviour, sleep) seems to be more successful in improving adiposity outcomes compared to single-component interventions (12–14,19–24). Involving parents was found to be essential in the prevention of childhood overweight and obesity, especially among children younger than 12 years (9,11,13,23,25–29). However, less than half of the earlier studies involved parents (25). Among the studies that did involve parents, three out of four demonstrated positive outcomes in reducing BMI or weight status.

Other characteristics that seem promising are:

- Combining educational components (e.g. targeting the children's knowledge, competences, literacy levels, and motivation) with environmental or structural components (e.g. free healthy school meals, smaller portion sizes, easy access to fruit and vegetables, physical activity as a part of the school day, free and/or easy access to leisure time activities, healthy space management of supermarkets, healthy alternatives in restaurants, decreased availability of fast food and sugary beverages, and healthy school policies) (9,13,16,28,29). The environment around the child, both at school and at leisure time (home and local community) must support healthy living.
- Targeting a multitude of the settings where children spend their time (23,30) and including multiple intervention components (9–16). Several systematic reviews have shown limited effects of interventions that are purely school based (31).
- Long-term interventions (twelve months or longer) appear to be more effective compared to studies of shorter duration (9,13).
- Co-creation and involvement of relevant stakeholders was also found to be an important factor in earlier, successful studies. This means involving stakeholders in identifying, prioritizing, developing, and implementing interventions using co-creative approaches and capacity building processes (32–37). (16,38,39).

4.3 Key components in prevention - diet, physical activity, screen time and sleep

Healthy diets and balanced energy intake are key factors in prevention of overweight in childhood, which must be addressed simultaneously in different settings for maximal effect, in a manner that supports food literacy, practical competence and long-term behaviour change via environmental changes. Danish children consume too little vegetables, wholegrains, and fish, and too much sugar and fat, compared to the Danish food-based guidelines and the Nordic Nutrition Recommendations (40–42). This worsens with age, which

means that preventive efforts are needed at an early age. There is considerable social inequality in dietary behaviours, as Danish children from households with short compared to long education consume less fruits, vegetables, dietary fibre, and fish and often skip breakfast (43). School food programs can improve dietary quality (44), and in Sweden, provision of free school lunches during basic school has been associated with long term effect on different indicators for better health and well-being, including education attainment, higher salaries, and higher final height later in life (45).

The diet of Danish children has been found to be less healthy during weekends compared to weekdays (46), and studies have shown that children primarily learn about eating behaviour, dieting, cooking etc. by watching their parents. Further, parents define their children's home food environment, e.g., access to healthy and unhealthy food items and whether there are family rules limiting the intake of unhealthy foods (47,48).

Physical activity (PA) is an important part of a healthy lifestyle for children, as it improves physical fitness, motor competences and well-being, and reduces the risk of developing overweight and non-communicable diseases later in life (49). Current recommendations are that children and adolescents should participate in at least 60 minutes of moderate-to-vigorous physical activity (MVPA) per day and 3x30 minutes of vigorous physical activity (VPA) per week. However, in Scandinavia and the rest of Europe, many children do not reach these targets (50,51).

A high percentage (about 70 percent) of 7-9-year-old children in Denmark participate in one or more club-organized sports. This contributes significantly to these children's MVPA and increases their chance of being active at a later age, when physically active free play during e.g., recess decreases. The participation rates, however, are much lower for children whose parents have low sporting experience and in families with limited socioeconomic resources.

Excessive recreational screen media use and short- and poor-quality sleep are important risk factors for the development of childhood obesity (52,53). While high screen use may be obesogenic by displacing engagement in PA during leisure time, it may also lead to insufficient sleep among children (54). Sleep is not only critical for learning, memory, and school performance. Inadequate sleep also disrupts hormone levels that regulate appetite and food intake (53). A third of Danish 6-11-year-old children use screen media >4 hours/day during weekends, and 24% of children in this age group report screen use just before bedtime on all weekdays (7,55). Furthermore, significant socioeconomic disparity is seen in children's screen media use, with higher prevalence of excessive recreational screen use and screen use just before bedtime in children from socioeconomic disadvantaged families. Insufficient sleep is also more common among children from families of low socioeconomic status (56).

In addition, we observe that more healthy screen use practices appear to be promoted by well-established parental rules of screen media use hygiene (when to use screen media, content- and amount of use), and we find a graded relationship of total recreational screen use, screen use just before bedtime and sleep time of children (57).

4.4 Generation Healthy Kids – the innovative approach

Generation Healthy Kids will build on the existing evidence, combining the characteristics and elements described above to improve the chance of success. The innovative aspects of the project are the combination of a randomized controlled trial targeting several important risk factors for excessive weight gain (dietary habits, physical activity, sleep, and screen time) with community capacity building and a systems approach (58). The unique combination is closely monitored for effect as well as process. The integrated intervention program will be unique in combining already tested effective intervention strategies with intervention components developed using co-creation and a systems mapping (58). Systems thinking will furthermore be used to ensure that a health equity lens is employed to understand the relationship between obesity and health inequalities locally (59). To reach children and families with low socio-economic status, focus will be on developing interventions that make healthy choices easy by creating healthy environments around the children, both at school and during leisure time.

4.5 The pilot study and the main trial

Generation Healthy Kids is a 4-year project including a pilot study which was conducted in January-April 2023, and a 2-year cluster-randomized controlled main trial to be conducted in August 2023 – June 2025. The present protocol describes the main trial.

The pilot study was approved by The Regional Committee on Health Research Ethics for Southern Denmark (Project ID 568021-22 S-20220059).

A thorough development and piloting is an essential prerequisite for ensuring feasibility and quality of the final intervention program to be assessed via the main trial. Based on the learnings and results from the pilot study, the final intervention program has been adjusted accordingly. Due to the timeline of the main trial, this protocol was originally submitted before the pilot study was conducted (protocol version 1.0, submitted 1 December 2022). Adjustments based on learnings and results from the pilot study were included in the **first amendment protocol** (protocol version 2.0 was submitted 12 May 2023 and was partly approved by the Committee on 6 July 2023; protocol version 2.2 provided response to the Committee's partial approval of 6 July 2023). The present **second amendment protocol**

(protocol version 3.0) describes some further adjustments to the study protocol prior to study initiation.

5 Study Objectives

5.1 Overall Objective

Generation Healthy Kids is a cluster-randomized school and community trial in which 24 local schools will be randomly allocated to intervention or control. The overall study objective is to investigate if a 2-school-year multi-setting, multi-component intervention focusing on healthy diets, physical activity, sleep and screen media habits in the school and local community can promote healthy weight and body composition in children who are in 1st and 2nd grade at inclusion (i.e., age 6–9 years at inclusion). We will also investigate the intervention's effects on dietary intake and nutritional status; food competences; physical literacy, activity levels, and fitness; sleep and screen media habits; growth; cardio metabolic health; cognitive and motor functions; school performance; and mental health and wellbeing. Furthermore, we will evaluate the implementation of the intervention.

In continuation of the above, we will investigate if the intervention can reduce social inequality in the outcomes, as well as explore potential effect modifiers such as sex, ethnicity, genetics, epigenetics etc. We will also explore associations between sociodemographic characteristics, health behaviours and outcomes cross-sectionally and longitudinally and use these data to validate and compare relevant measurement tools.

The primary outcome is fat mass (FM) in kg. We hypothesize that the combined intervention components will counter the development of increasing overweight and unhealthy weight with age and thus will cause less FM gain in the intervention group compared with the control group.

5.2 Specific Objectives

5.2.1 Main objective

The main objective is to investigate the impact of the 2-school-year intervention program on development of FM in the intervention group compared with the control group.

5.2.2 Secondary objectives

As secondary objectives, we will investigate the impact of the intervention program after 1 and 2 years on:

- Other body composition measures, including fat free mass (FFM), FFM index, FM index, FFM-to-FM ratio, % FM;
- Anthropometric measures and growth, including BMI z-score, prevalence of obesity, overweight, normal weight and underweight, waist circumference, linear growth and growth-related biomarkers;
- Cardio metabolic health, including blood pressure, blood lipid profile, insulin resistance markers;
- Other health related outcomes, including inflammation;
- Cognitive and motor functions, physical literacy, and school performance;
- Mental health and wellbeing;
- Dietary intake, biomarkers of food and nutrient intake and status and appetite and food competence;
- Physical activity, physical fitness, leisure-time activities, sleep- and screen media habits;
- Long-term (>2 years) body composition based on height and weight obtained from routine data in The National Child Health Register (Børnedatabasen).

Also, the collected data will be used for validations of associations between biological mechanisms and various measurements and outcomes (data analysed as observational data e.g., based on baseline data, longitudinal data or using the data from one group only), as well as the impact of background characteristics, such as socioeconomic position, family characteristics, sex, ethnicity, genetics, and epigenetics etc. on the effectiveness of the intervention.

A specific objective of Generation Healthy Kids is to involve relevant local and national partners in the development and implementation of the multi-component intervention program, and to create local engagement and ownership for the implementation of the intervention. The project will work with multiple public, private and non-governmental organizations in the local community, including leisure time- and sports organizations, supermarkets, restaurants, and fast-food chains, to create healthier environments, e.g., by increasing access to healthy foods in supermarkets. The effects of such initiatives will be monitored and evaluated by e.g., sales data and measures of shelf-space allocation from supermarkets.

In addition, an objective of the study is to understand and document implementation-related factors, causal mechanisms and contextual factors which shape the intervention's outcomes. Therefore, a thorough process evaluation will be conducted, see section 6.5.4.

A protocol résumé is found in Appendix 1.

6 Investigational Plan

6.1 Study Design

The study will be designed as a community-based open label cluster randomized superiority trial with two arms (intervention and control). Individual schools will form the basis of the cluster. A comprehensive list of all schools will form the school sampling frame, which is further explained in section 6.2.4. Teachers and pedagogues at each school will have a key role in implementing, coordinating, and anchoring the intervention.

Geographically, the 24 schools are located in either DK-East, which includes Region Zealand and the Capital Region of Denmark and is mainly administered by University of Copenhagen (UCPH), or in DK-West which includes The Region of Southern Denmark and is mainly administered by the University of Southern Denmark (SDU).

6.1.1 Justification of study design

The study is designed as an open label cluster randomized trial as the primary objective of the study is to examine the effectiveness of the Generation Healthy Kids Program on the primary and secondary outcomes specified in sections 5.1 and 5.2.

6.1.2 Randomization

In this cluster-randomized trial, randomization will occur at school level and will be performed prior to inclusion of participants (i.e., children) and collection of informed consent. A stratified block randomization will be carried out in a 1:1 ratio. To prevent baseline imbalance of cluster characteristics across the two study arms, we will use covariate-constrained randomization (60). The covariates will be at school- and school district level with tentative factors being number of classes per school year (at least two classes per school year required); the socioeconomic profile of the school based on parental educational level (proportion of parents with a shorter education); ethnicity of the children (proportion of pupils with non-Danish origin in the respective school); and degree of urbanization of school district. The randomization procedure will be carried out by a statistician not otherwise involved in the project (i.e., not involved in recruitment of schools or participants), and the procedure will be concealed to the investigators.

6.1.3 Timeframe of the trial

The trial will run over two school years (2023-24 and 2024-25). Baseline data will be obtained before the start of the intervention, and follow-up data will be collected during the two school years period.

6.1.4 Sample size justification

The sample size calculations are based on the primary outcome for the study being FM (kg) at 18-22 months as measured by air displacement plethysmography (BODPOD). For the power calculation, a mean cluster size of n=58 children is assumed, which we expect based on the current status of recruitment of schools. We anticipate a total of 2,350 eligible children and a participation rate of 70%, and with an expected drop-out/missing data rate of 15%, which provides an expected mean of 58 children in each of the 24 clusters in the analytical sample. Others and we have managed to limit missing data to approximately this amount in previous large-scale school-based cluster randomized trials in young people (61–63). Thus, we aim to enrol a mean of n=69 children from each school. We assume a cluster size coefficient of variation of 0.24 based on the OPUS School Meal Study (64) PHASAR data (65) (1st to 4th grade children), a standard deviation of FM of 4.65 kg in both groups based on OPUS, and a conservative estimate of the intra-cluster correlation coefficient in FM (kg) of 0.03 based on PHASAR data (PI´s of the OPUS and PHASAR study are part of the scientific team in Generation Healthy Kids). Finally, because we plan to adjust for baseline FM (kg) in our primary analysis, we also assume a correlation between baseline and follow-up FM (kg) of 0.8 in the power calculation. With an expected enrolment of 24 clusters in 1:1 ratio allocation to intervention and control, we have decided to additionally control for type-1 error due to a planned correction of small (cluster) sample using the Satterthwaite correction for mixed linear models (66) by lowering alpha to 0.025 in the power calculations. With a cluster size of 58 and 24 clusters (expected n=1,398 children in the analytical sample and 1,645 enrolled children at baseline), we have a power of 80% to detect a significant mean difference of -0.81 kg FM or greater at 18-22 months between intervention and control. In OPUS data, this difference corresponds to -0.15 BMI-z score (95% CI -0.15; -0.17), and a previous cohort study based on The Copenhagen School Health Records Register reported that a 1 kg increase in FM at 10 years of age was associated with a 12% and 15% increased risk of type 2 diabetes at 50 years of age for boys and girls, respectively (67), which corresponds to a 10% and 12% decrease in risk per -0.81 kg FM. Thus, the planned study is powered to detect a fairly small, but also clinically relevant size of mean difference in FM at 18-22 months between intervention and control.

For the blood sample outcomes, we expect 12 clusters (DK-East) with an expected 1175 children being eligible to participate. As we expect a 50% participation rate for blood sampling and 15% missing data rate, the mean cluster size will be 42 children in the analytical sample. With this cluster size, 12 clusters, and assuming a correlation between baseline and follow-up HDL cholesterol of 0.65, a standard deviation of 0.31 mmol/L in both groups based on OPUS data we have a power of 80% to detect a significant mean difference of 0.10 mmol/L HDL cholesterol or greater at 18-22 months between intervention and control (alpha=0.025).

6.1.5 Blinding

The study will be open label, as it will not be possible to blind participants due to the nature of the intervention. Also, it is not possible to blind personnel delivering the intervention (i.e., teachers). Outcome assessors will not be blinded, as we judge this to be impossible to implement with success. However, the primary outcome (FM) is objectively assessed not involving judgement by the assessor during the assessment and therefore unlikely to be influenced by knowledge of intervention allocation.

6.2 Study Participants

The study population will be approximately 1,600-1,700 children in 1st and 2nd grade (i.e., age 6–9 years at baseline) who attend 24 schools in DK-East (Capital and Zealand Region) and DK-West (Region of Southern Denmark).

We have selected young school children as the target group for the intervention, because the prevalence of overweight and obesity increases from early childhood until adolescence, from approximately 12-13% at ages 6-7 years to approximately 18-19% at ages 14-15 years (68–72). Furthermore, early childhood is an important time period for formation of habits in relation to diet and physical activity which may persist into adulthood (73). Thus, early intervention is crucial to prevent unhealthy weight development.

6.2.1 Inclusion Criteria

All children attending 1st or 2nd grade in schools recruited into the study will be eligible to participate.

6.2.2 Exclusion Criteria

There are no exclusion criteria for the children. If a child has food allergies, plaster allergies, or chronic disease (Appendix 2A and 2B) that cannot be accommodated in parts of the interventions or measurements, or if the parents, teachers, or research team judge that a child has physical or mental disabilities which cannot be accommodated in parts of interventions or measurements, the child can participate in the remaining parts of the program.

6.2.3 Withdrawal Criteria

The parents will be informed that they can withdraw their child from the study at any time and for whatever reason without any explanation or consequences for the child's daily life at school. If parents withdraw their child, they will be asked if they are willing to explain the reason, but responding is optional, i.e., entirely voluntary (see Appendix 3). In case of participant withdrawal, the data collected up to the point of withdrawal will be used in data analyses. Withdrawn participants will not be replaced.

The Primary Investigator and Clinically Responsible Physician (independently of each other) have the right and obligation to terminate participation of any child at any time if it is deemed to be in the best interest of the child, e.g.:

- If a child’s safety or well-being is compromised by further participation
- If the investigator judges that the child does not want to continue his/her participation

Or in the interest of completion of the study, e.g.:

- If the child does not comply with the study procedures in a way that impacts the scientific integrity of the study.

In this case, reasons for termination of the child’s participation will be recorded, and the child’s parents will be informed about the decision.

Dropouts and children who do not participate in the study will not be excluded from participating in activities which are incorporated as a part of the school’s regular schedule and activities, including physical activity during school time, school meals, and learning activities during school time regarding screen media habits, sleep habits and food competences. However, no data will be collected from these children.

6.2.4 Recruitment of schools

The sampling frame of the study will be constructed based on information on all schools in Denmark obtained from The Danish Ministry of Children and Education data-warehouse. Information obtained at school-level will include the number of children attending each school in 1st and 2nd grade. Special schools and schools in areas that already take part in similar research projects will not be eligible for participation. An initial sampling frame list will prioritize schools with at least two classes per year group and relative geographical proximity to the study centres. This sampling frame list will be expanded if the success of recruiting schools is lower than expected.

The schools selected to be part of the sampling frame will be contacted directly via the school leader and/or the head of the school board, and the school will be invited to participate in the study. Furthermore, municipalities will be contacted to assist with recruitment of schools and to ensure overall support for the study. Finally, in addition to this direct recruitment procedure, an open advertisement for the study will be placed on relevant platforms (e.g., Folkeskolen.dk and LinkedIn) to recruit potentially interested schools for the study.

6.2.5 Recruitment and enrolment of study participants

6.2.5.1 Invitations

Families in the selected schools and classes will receive invitations to the study through Aula and in printed form from teachers. Aula is an online communication platform used by all schools and approved for the processing of sensitive information.

The invitation message on Aula will include:

- An invitation letter, including an invitation to information meetings about the study at the school (Appendix 4A and 4B) and an electronic link to sign up for the information meeting;
- Written participant information about the study (Appendix 5A and 5B);
- The pamphlet '*Forsøgspersonens rettigheder i et sundhedsvidenskabeligt forskningsprojekt'* reviewed and published by the ethical committee system, Jan 2023, will be included on the last page of the participant information.
- Information about processing of participants' personal data and biological material in accordance with the European General Data Protection Regulation (GDPR).

Parents (or custody holders) are asked to carefully read all the information material and to explain the content to their child/children. The information material will be translated into the

languages judged by the schools and research team to be most relevant for the included classes.

Parents sign up for information meetings at the school via a secure online system. Parents are informed that they must bring their child to the information meeting, and that they can also bring an assessor (”bisidder”) if they wish.

Prior to the information meetings, members of the research team will visit the schools in the morning during the time slot where children are usually dropped off at school, and/or in the afternoon when children are usually picked up or at other times considered relevant by the school. The researchers will hand out one-page information leaflets about the study (Appendix 8A and 8B), encourage parents to participate in the information meetings, and answer any questions from parents about the study. In addition, one-page information leaflets will be handed out to parents in the involved classes by research team members and school staff, and posters about the project will be posted at the schools (Appendix 9).

Members of the research team will also visit the involved classes during school time and inform the children about the study activities in a language and at a level appropriate for their understanding, cf. Committee Act § 5, stk. 2. The parents and children will always be informed that participation is entirely voluntary, and that the decision on whether to participate will in no way impact the child’s schooling.

6.2.5.2 Information meetings for families at the school

The information meetings will be held at the school in groups of maximum 25 families per room. Parents will be informed that they must bring their child to the information meeting.

At the meetings, the background and objectives of the study will be presented by the Investigator, or by a qualified person who is delegated to give the information, and the parts of the study that involve the family and child will be explained in detail to both the parents and the children (see the content of the information meeting in “Table 1. Overview of study procedures and activities“). Short videos illustrating the different measurement procedures, (e.g. the BODPOD, accelerometer, measurement of blood pressure and blood sampling) may be presented (Appendix 27A-D). Also, the parents will be informed that in case of an incidental finding concerning their child, e.g. hypertension, they will be contacted either by phone or e-mail if it is judged to be clinically relevant by the Clinically Responsible Physician.

In line with the Committee Act § 5, stk. 2, information to the children will be provided in a language and at a level that is appropriate and suited for the children’s age and comprehension. The information to the children will be provided by project staff experienced in communicating to children or who have been trained specifically for this task. There will be

time and room for asking questions, also in a separate room one-to-one. If the parents or the child are unable to participate in the information meetings, they can have the oral information for themselves (see below section 6.2.5.4). If the project staff judges that some parents need a second explanation of the study, this will be encouraged. It will be emphasized that the participants have the right to at least 24 hours consideration time, that participation is voluntary, that the participants can withdraw from the study at any time without giving a reason and without any consequences, and that all personal data will be kept strictly confidential. It will be ensured that parents with insufficient knowledge of Danish will be adequately supported to fully understand the information provided.

The research team will register which parents and children are present at the information meetings, and during information provided to children in the classroom, in order to make sure that the custody holders and the child have received thorough oral participant information and fully understand the study and its procedures.

6.2.5.3 Informed consent process

Parents/custody holders will only be able to provide written informed consent if *1)* the parents have received both written and oral participant information in accordance with the Committee Act § 5, stk. 1, and *2)* the child has received oral participant information about the study in accordance with the Committee Act § 5, stk. 2. All parents/custody holders are informed that they are entitled to a consideration period of minimum 24 hours.

In case the parents/custody holders and the child want the child to participate and do not wish further consideration time, both parents/custody holders together with investigator or a delegated and qualified person fill out the paper version or an electronic version of the consent form (Appendix 6A and 6B, Standard Forms S6 and S5) at the information meeting. The electronic consent form will be accessible via a link to RedCap (see further below in section 6.2.5.4). Parents will also be informed that by signing the informed consent they give permission for the controlling authorities to access their data, and that a person carrying out these functions is subject to the rules of confidentiality.

In the supplementary information about GDPR, parents/custody holders and participants will receive additional information about the way data and biological material are collected, shared, and made available for other researchers and data processors in compliance with GDPR.

If parents/custody holders or children wish further time for consideration, the parents/custody holders will receive a link to the electronic version of the informed consent form. This will be sent via Aula or a personal e-mail address or phone number they provide to

the research personnel. The informed consent form must be signed by the parents/custody holders and the investigator or a delegated and qualified person, and any additional questions from the parents must be answered. No study procedures will be initiated before the informed consent is obtained.

One parent or custody holder can empower the other parent or custody holder to decide whether the child should participate (Appendix 7). An electronic solution for this will also be made available to the parents/custody holders (Appendix 7).

As the study participants are under the age of 18, they cannot give informed consent. However, the project staff will at all times during the study be aware of the child's response and reactions to the study (both verbal and non-verbal responses); if the project staff at any time judges that the child does not want to participate in the study, the child will not be included.

6.2.5.4. The electronic consent form

Parents/custody holders can fill out the consent form (Appendix 6A and 6B, Standard Forms S6 and S5) electronically via a link they receive via Aula, or via a personal e-mail address or phone number they provide to the research personnel. They can sign the electronic consent form by drawing their signature in the designated field. After signing the form, data is encrypted and sent to a REDCap server at the researcher service Odense Patient Data Explorative Network, and a static copy of each completed form will be stored in REDCap ensuring that the forms cannot be edited. A copy of the completed form will also be sent directly to the participants' e-mail address.

The identity of the parent/custody holder who received the link to the electronic consent form is verified via the Aula platform where parents are verified via NemID/MitID upon their first login or in person.

6.2.5.5. Parents who do not participate in information meetings

Follow-up information meetings, either in groups or as individual meetings, online or face-to-face, will be arranged for parents/custody holders and children who were not able to participate in the initial meetings.

School staff will be asked to encourage families to participate in information meetings to learn about the study, but it will always be stressed that participation in the study is completely voluntary, and that a decision not to participate will in no way impact their child. We will ask school staff (e.g., teachers and pedagogues) to offer parents the possibility to be contacted by

telephone by a member of the research team, if the parents wish further information about the study before deciding whether to participate.

6.2.5.6. New pupils enrolled in the schools during the study period

If new pupils are enrolled in the participating school classes during the intervention period, they and their parents will be invited to participate in the study. Parents will initially be contacted by the schoolteacher via Aula or in person. If they are interested in hearing more about the study, the parents and children will receive oral and written participant information by the research team following the same procedures as described above in section 6.2.5.5. Written informed consent will be obtained from custody holders before enrolment into the study, and the parents will be asked to fill out appendix 2A or 2B about allergies, chronic diseases etc. at enrolment.

6.2.5.7 Recruitment of local stakeholders

For eight of the 12 intervention schools, an expanded local community intervention will be performed (see section 6.3.4 for further details). In these local intervention communities, a stakeholder analysis will be done together with representatives from the municipality and school. Potentially relevant stakeholders will initially be invited for a bilateral meeting. Relevant stakeholders will initially be defined as community leaders who have authority to initiate action within child health and well-being, including leaders from the municipality, the local school, local sports- and leisure time organizations, and private businesses such as local supermarkets and restaurants. These leaders will build a system map of drivers of obesity in the local community as further described in section 6.3.1. The system map captures drivers of childhood obesity in the local community. Subsequently, a larger group of community representatives will be engaged from organizations whose activities and agenda includes remit and capacity to influence children's food and activity environments, screen time and sleep. Furthermore, selected local leaders and stakeholders in each community will be invited into a Local Community Partnership Group (LCPG) that will function as a steering group during the project period and help ensure coordination and long-term local anchoring of the intervention program.

6.3 Intervention

6.3.1 Principles of the intervention

The development of the intervention in the intervention schools will be guided by the Medical Research Council framework on designing complex interventions as well as a systems thinking

approach (58,74,75). Furthermore, the super-setting approach will guide the process of creating synergy between intervention components (76).

The development of the intervention will take place on three levels:

a) Core intervention components at school level based on existing evidence
b) Intervention components developed in co-creation with national stakeholders
c) Community capacity building in selected intervention local communities

The core intervention components at school level are pre-defined based on existing evidence and will be described in section 6.3.2 Intervention in schools and after school clubs. The intervention at school level will be refined based on experiences from the pilot study. Furthermore, the intervention components will be adapted to the local context of each school.

Other interventions will be developed primarily outside school, focusing especially on changing the environmental drivers of childhood obesity (social, cultural, physical, economic, and political). Inspired by the Public Health England's publication "Whole systems approach to obesity: A guide to support local approaches to promoting a healthy weight" (59), a co-creation process with national stakeholders took place prior to the pilot study to develop relevant interventions. The national stakeholders included representatives from national supermarket chains, fast food restaurants, Rådet for Sund Mad [Council for Healthy Food], national leisure time organisations, Local Government Denmark (KL), The Danish Healthy Cities Network, Danish Health Authority, The Danish Food Administration etc. After the initial stakeholder analysis, the process of co-creation consisted of a series of bilateral meetings and two workshops. In the first workshop, a systems map was developed using the software STICKE (77). In the second workshop, stakeholders came together to prioritise areas to intervene in the local system and propose collaborative and aligned actions to bridge interventions between settings. The project drew on the expertise and resources of The Intersectoral Prevention Laboratory (www.forebyggelseslaboratoriet.dk) who is responsible for the process of facilitating the creation of a systems map using STICKE.

Community capacity building will take place in some of the local intervention communities (eight of the intervention schools, located in six different municipalities). Initially, a stakeholder analysis will be conducted to identify community leaders and stakeholders that have authority to initiate action and that would be relevant to engage in an intervention anchored in the local community promoting healthy weight among local children. Inspired by the cluster-randomized trial, WHO STOPS (78), the process will consist of the phases listed below:

- Collecting and communicating local data and scientific evidence to raise local awareness of childhood obesity and to engage and recruit community leaders.
- Identifying, inviting, and working with a smaller group of community leaders who have authority to initiate action, including leaders from the municipality, the local school, local leisure time organisations, and private businesses including local supermarkets and restaurants. These leaders will build a causal loop diagram of the causes of childhood obesity in their community (79) using STICKE (77).
- Engaging a larger group of community representatives from organizations whose activities and agenda includes remit and capacity to influence children's food and activity environments, screen media habits and sleep.
- Involving the larger group of community representatives in working together to design actions to prevent childhood obesity that they can carry out across the community, inspired by the causal loop diagram or systems map and informed by a prepared evidence brief on obesity prevention, including case studies from previous successful interventions.
- Ongoing data collection and updates of the systems map to enhance implementation and diffusion of the selected actions and stimulate new ideas in a constructive, capacity-building cycle.
- Selected local leaders and stakeholders in each local community will be invited into a LCPG that will function as a steering group.

To develop an overall coordinated intervention programme, the different intervention components developed at different levels and in different settings will be combined using the Super-setting Approach (76). To promote integration and synergy between interventions, common themes for all settings (e.g., mental well-being, taste and senses etc.) will run throughout the intervention period for 2-3 months each.

6.3.2 Interventions in schools and after-school clubs

Prior to initiation of the intervention and measurement schedule (cf. section 6.6.), project staff may visit the school classes and explain the study activities to the children during school time in their classroom. This will occur at a level appropriate for the children's age group by project staff specifically trained for this task. Children whose parents have not provided informed consent to participate in the study will be exempted from this information, unless it is relevant for all children (e.g. if it relates to the school meals that will be offered to all children in the classes, regardless of participation in the measurement schedule).

School management and school boards at the intervention schools will be offered counselling and guidance on developing and implementing school policies within the project's focus areas,

e.g. school policies on digital devices, physical activity during the school day, and school dietary policies.

6.3.2.1 Food and nutrition

The dietary intervention will be based on the national Danish food-based dietary guidelines (40) and the Nordic Nutrition Recommendations (41) and seeks to promote a balanced energy intake, increased healthy food intakes, and improving children's healthy food competences. A key focus will be on increasing intakes of wholegrains, fruits, vegetables and legumes, and fish, reducing the intake of sugar, and on drinking water.

The dietary intervention in school will include pre-prepared do-it-yourself lunch, consisting of for example rye bread sandwiches, pasta salad, raw vegetables etc. 4 days per week in most school weeks. The children will be involved in the table setting and cleaning after the meals. We expect that extra time will be needed to be scheduled for eating by the schools. Lunch will be offered to all children regardless of their participation in the measurement schedule and will be free of charge. We will collect information about allergies and other dietary considerations from the parents of participating children at enrolment (Appendix 2A). Parents will be informed that the meals cannot accommodate special dietary needs and that the menu with full list of ingredients (including allergens) will be posted on a specific website one week before, for them to check which parts their child can eat. The teachers/pedagogues will help handle allergies in the eating situation as in a normal school day. We will ask the teachers to inform us about food allergies in children who wish to have the meals, but who are not participating in the measurements. To encourage water consumption, cold-water dispensers will be made available if this is feasible.

In addition to this, we will provide tips and inspiration materials for the parents on how to make easy, healthy midmorning snacks, and if relevant also lunch packs. We will also develop fun and engaging materials and hands-on food competence exercises that the teachers can use in their teaching to support the children's knowledge, self-efficacy and liking of healthy foods as well as practical skills in food preparation. Examples are videos about the food served, vegetable rhyme books, sensorics taste exercises, vegetable puzzles and following a recipe to cook a simple dish together in the school kitchen.

In the after-school clubs (SFOs), we will work with their existing offers of breakfast and afternoon snacks and provide tips and inspiration to encourage the staff to follow the national food-based guidelines. Other activities focusing on healthy diet may be arranged in

collaboration with the SFO, such as afternoon café for parents where children cook and serve healthy snacks or meals and/or excursions to local farmers, greengrocers, or fishmongers.

6.3.2.2 Physical activity

The amount of moderate and vigorous PA during school time will be increased through three approaches: a) 3x40 minutes of organized vigorous PA, b) education of teachers and c) improved facilities and structure for PA during recess and in after school clubs.

a) 3x40 minutes per week of organized vigorous physical activity during school time

To ensure that all children reach the recommended 3x30 minutes of vigorous activity per week, we will implement three weekly 40-minute sessions in the school curriculum. These sessions will be carried out throughout the study period and will encompass modified and varied sporting activities (80) and games, which promote motivation, active involvement of all children, and intensity. These 40-min sessions are developed to promote joy though play, provide appropriate challenges of the children's motor competences and physical capacity and, importantly, to achieve at least 30 minutes of varied, vigorous activity in relation to cardiopulmonary and musculoskeletal loading for all children, irrespectively of sex, fitness levels, skills, and prior experience with sporting activities (80). The activities will thus be adapted to allow participation for all children.

b) Physical activity during school recess

In primary schools, recess time amounts to almost 5 hours weekly and is a setting where young school children on average have a high level of PA (81). This makes recess an important part of the total daily and weekly amount of MVPA for young school children. It has been shown that improved facilities, both in terms of permanent play and sport facilities (e.g., climbing frames, football goals) and loose play equipment (balls, skipping ropes, skateboards), increases mean daily amount of PA among young school children as well as the proportion reaching daily recommended levels of MVPA (82,83). This part of the integrated PA intervention centres on distributing packages of loose play and sports equipment to school classes and making agreements with schools and teachers about how these are distributed and collected at the beginning and end of recess. Previous projects such as Move@School have shown this to be feasible and a popular approach for improving children's opportunities to be active during recess. This will be supplemented by the concept of drawing lines in the schoolyards for 4-square and similar games and, if relevant, providing small goals for basketball, floorball and football.

c) Physical activity in after-school clubs

Danish school children at age 7-8 years spend about 10 hours a week in after-school clubs. Mean activity level is moderately high, but great variation is seen between children (81). More

physical activity in this setting will be facilitated by implementing policies limiting computer gaming as an activity and facilitating outdoor activities as much as possible. In collaboration with the staff at the after-school clubs, activities suiting the institutional facilities and time structures will be developed and the equipment needed will be identified.

6.3.2.3 Sleep and screen media habits

During the study period, teachers/pedagogues will be asked to facilitate small assignments on screen media and sleep practices with the children during school time. The children will be asked to discuss and reflect upon screen media and sleep practices in their family, based on material from e.g., Medierådet (The Danish Media Council). The results of the children's assignments will be presented at later workshops for the parents (see below in section 6.3.3.2). This is done to integrate the children's perspective in the parent workshops, especially in relation to screen media habits.

6.3.2.4 Education of school staff

School staff (teachers and pedagogues) involved with the 1st and 2nd grade classes at the intervention schools will be asked to participate in three courses in the beginning of the study period:

Course on food and nutrition (approx. 4 hours): Teachers and pedagogues involved in preparation of the school food and having lunch with the children will receive education and material in e.g., the hygienic and pedagogical aspects of food and eating, nutrition, sensorics as well as tips and exercises to be used in the classroom.

Course on PA (6 hours): The aim of the educational program for PE teachers and other teachers with responsibility of PA in the class is to develop children's understanding, motivation, and abilities for being active in different sports and exercise activities throughout life. In many ways, the aim is to develop children's physical literacy (PL). We aim to help schools and PE teachers fulfil the ambitious aim of PE by offering a short educational program developed and tested in the PE component of the Y-path program (84). PE teachers will be offered this short education in combination with peer supervision and sparring on how to support children's PL development and how to create a motivating and inclusive climate for all.

Course on children's screen media and sleep habits (3 hours): Teachers and/or pedagogues with overall responsibility for the class will participate in an educational program related to children's sleep and screen media habits. The educational program will inform teachers about evidence and recommendations on sleep and screen media habits, and examples of class exercises and activities for the workshops will be presented.

6.3.3 Interventions focused on the child through the family

The interventions focusing on the child through the family will include fixed intervention components determined à priori by the research team, as well as co-created intervention components developed in collaboration with families.

During the intervention period, up to four parent or family workshops will be held at the schools each year. The workshops will be used to convey important scientific knowledge within each of the project's focus areas (diet, physical activity, screen media habits and sleep) to the parents. The workshops will also include practical exercises, such as inspiration for ways that parents can engage in physically active play with their children and/or tips and tricks for involving children in daily cooking. Furthermore, the workshops will be used to ask parents to provide their views on perceived individual-level and structural barriers for adopting healthier habits, and in collaboration with parents, we will co-create interventions which could support behavioural changes within each focus area.

The form of each workshop will be decided in collaboration with the school. The workshops will be facilitated by members of the research team, teachers, pedagogues and/or external experts (such as Medierådet, Børns Vilkår, Hello Kitchen etc.), depending on the content of the specific workshop. Participants in the workshops may be parents alone and/or parents and children together.

We will pay special attention to avoid lecturing, parent bashing, stigma, and unhealthy focus on body weight. Focus will be on actively engaging and involving parents in promoting healthier lives in their family and in the local community. To reach all parents irrespective of ethnic and educational background, we will provide simple messages supported by visuals, and we will translate material into numerous languages if recommended by the school.

Written study material, such as invitations for workshops and inspirational material, will be sent to parents by e-mail via the software REDCap and/or via AULA and in print via the teachers. Inspirational material may include, for example, inspiration for birthday parties and playdates including fun physical activities that promote movement as well as suggestions for simple, fun and healthy food which we will develop together with the school and/or other stakeholders. The planned intervention components which involve the families within diet and sleep/screen media habits are described in more detail in the following sections.

We will seek to actively include the children's own perspectives in the development of certain intervention components. This will be done by conducting interviews and focus groups (class-wise or in smaller groups) with the included classes in order to obtain the children´s perceptions of what constitutes a "healthy" life as well as barriers and facilitators for healthy

living. The data collection process will be guided by participatory action research methods, and we will use the research method "Future Workshops" where children are encouraged to establish their vision for a healthier school and local environment (112). We will seek to integrate the ideas and visions put forward by the children into the overall intervention program, where this is realistic and feasible.

6.3.3.1 Diet

At least one of the parent workshops held at the school during the intervention period will be used to provide motivation, tips, and strategies for healthier eating in the family. If feasible, there may also be cooking workshops for the families at the school, where families will be invited to cook with their children following simple and hands-on recipes to gain practical healthy food competences in the families.

6.3.3.2 Screen media habits and sleep

Based on the current knowledge of determinants of screen media habits in children, as well as our experiences in the family-based 'SCREENS trial' (57) and the ongoing school-based 'Screen-Free Time with Friends', the intervention will mainly focus on the interpersonal- and institutional (school) level, i.e., the family and peer social networks (85,86).

The overall aim of the screen media habits and sleep intervention component is to promote healthy screen media habits, encourage healthy sleep practices in the participating children, and to reduce the gap between the parents' and child's understanding of digital literacy.

The intervention will contain fixed generic components such as facilitating class culture and providing parents with the latest knowledge and recommendations within sleep hygiene and screen media habits.

Inspiration sheets

All parents, (also those who have not provided consent on behalf of their child to participate in the study), will receive inspiration sheets introducing knowledge and recommendations about screen media habits and/or sleep, potential tools to address this knowledge as well as suggestions for child and parent activities to support dialogues about sleep and screen media habits between the children and parents. Through these inspiration sheets we seek to encourage parents to reflect and consider the sleep and screen media habits in their family. We will provide parents with tools they can choose to use to support the introduction and maintenance of e.g., screen media rules. Importantly, any specific screen media rules will be determined by the parents/family and not by the researchers.

Parent workshops on sleep and screen media habits

Several parent workshops will be held over the two-year intervention period. The workshops will include recommendations, information, and dialogue focusing on children's sleep and screen media practices. The workshops will focus on the assignments on sleep and screen media habits that children completed during school hours (see 6.3.2.3).

The goal of the workshops is to increase parents' awareness of their own and their children's screen media practices, strengthen the dialogue with other parents, their own child, and between children within the provided subject (e.g., evening screen media use).

The topic for the workshops is planned to be an introduction to the knowledgebase for the importance of better sleep and balanced screen media habits. The workshops will also focus on the exchange of experiences (pros and cons) with sleep and screen media habits at home and during child playdates among parents and reflections on potential strategies to support better sleep and screen media practices among the children during leisure time periods, in the family, and to increase the awareness of alternative leisure time activities (e.g., participation in after-school clubs, sports clubs).

All parents will be invited to participate in the workshops even though they have not consented on behalf of their child to participate in the study.

6.3.4 Intervention focused on the local environment

The community intervention will be conducted in the local community surrounding eight of the 12 intervention schools (these eight schools are located in six different Danish municipalities).

For four of the 12 intervention schools, there is a control school located in the same municipality. In order to avoid contamination between clusters, these four intervention schools will not receive the local community intervention.

6.3.4.1 Local supermarkets, restaurants, and fast-food outlets

To promote healthier eating, children and families need to be supported within their local community by food environments that make the healthy choice the easy choice and not a challenge in their everyday life (87–92).

Within the national partnership established in connection with the study (see section 6.3.1.), new solutions will be developed to promote healthier eating among children, e.g., decreasing portion sizes, improving the availability of fruit, vegetables, cold fresh water, and healthy snacks, and decreasing the availability of sweets and sugar-sweetened beverages in supermarkets, restaurants, cafés, fast-food outlets and local sports clubs. Priority will be given to increasing the availability of fruit and vegetables, including convenience products, to make cooking with vegetables easier. The local community partnership groups (LPCGs) established (see section 6.3.1) will have an important role in adapting and anchoring the interventions in the local setting.

Furthermore, stakeholders from each of the selected local intervention communities will be invited into a co-creation process and to join the LCPGs. Invited stakeholders will be representatives from local sports organizations and other leisure time organizations for children, the school, the municipality, local supermarkets, restaurants and other private companies, parents, and other relevant local actors. The LCPGs will be responsible for developing and implementing activities within the local community that can promote healthier environments for children and families, including healthier food environment, easier access to active living including participation in sporting activities and outdoor activities, community-building activities etc.

6.3.4.2 Sports organizations

To support children that are not in sports clubs to join and become members, we will establish collaborations between schools, municipalities, and local sports clubs. Such collaborative initiatives have been shown to benefit families with low sporting experience, as these families are more positive towards sporting activities initiated via the school. Collaboration with sports organizations will occur in all 12 intervention schools, e.g. collaboration on establishing active summer camps during the summer holiday.

6.3.5 Compliance

Adherence to the intervention components will be explored at different levels using multiple method e.g., interviews, questionnaires, observations, logs, accelerometry, dietary registrations and biomarkers. As part of the evaluation of the implementation, we will assess whether the different intervention components are implemented by intervention providers, e.g., teachers and other pedagogical staff, as intended and to what extent (dose delivered). Moreover, we will explore to what degree children and parents are exposed to the intervention component (dose received and reach). We will also explore participation in general.

6.4 Control schools

The control schools will be offered education related to physical activity (FIT FIRST teen) for teachers of the eldest classes in the school, and all children in the classes participating in the present study will be offered participation in an activity day by the end of the study period. Also, as in the intervention schools, participating children will receive a small gift at each measurement round (see section 9).

6.5 Overview of study procedures and activities

The table below summarizes the activities described above and provides an overview of the planned study procedures as well as measurements, which will be conducted in 3 test rounds i.e., at the beginning of the first school year and at the end of each of the two school years. The measurements will be further detailed in section 6.6.

Table 1. Overview of study procedures and activities

**August–September 2023**	**Initial contact**<br><br>• Invitation letters and participant information are distributed to parents / custody holders
**August–October 2023**	**Information meetings at the school**<br><br>• An understandable presentation of the context and purpose of the study<br>• An understandable explanation of the study interventions and measurements to parents and children.<br>• Description of rights and considerations before decision<br>• Description of foreseeable risks, adverse events, complications, and inconveniences<br>• Unpredictable risks<br>• Voluntary participation and right to withdraw at any time<br>• Review of pamphlet "Before you decide" (11.NVK marts 2019)<br>• Information regarding handling of personal data / biological material, confidentiality, and general compliance with The European General Data Protection Regulation / Databeskyttelsesforordningen.

<table>
<tr><td></td><td>

That all data collected during the participation in the study will be used in the event of a participant chooses to withdraw from the study
The right to time for reflection
Questions
Explanation of informed consent and withdrawal
Collection of informed consent (including the possibility for 24 hours of consideration time)
Evaluation of inclusion/exclusion criteria including the allergy questionnaire

Staff education

Courses at schools – an education program for teachers and related staff involved in the intervention (see section 6.3.2.4)

Preparation of measurements

Instructions on dietary recording and accelerometer measurement

</td></tr>
<tr><td>Round 1: September-November 2023 (baseline)

+

Round 2: April-June 2024

+

Round 3: April-June 2025 (endpoint)</td><td>
Measurements at home:

Three-day dietary record, app. 30 min/day
Accelerometry
Screen/app time
Questionnaires
Adverse events

Measurements in school:

Fasting blood sample and time for breakfast (only children in DK-East, see below section 6.6.3.3)
Height, weight, and waist circumference
Body composition (BOD POD and bioimpedance)
Blood pressure and resting heart rate
Mounting of accelerometer device
Cognitive functions and school performance (math and reading)

</td></tr>
</table>

	• Physical and motor function tests (handgrip strength, balance, vertical jump, sprint, agility, gross and fine motor function)<br>• Yoyo running test<br>• Questionnaires
**October 2023 to June 2025**	**Activities in school and in the community**<br><br>• School lunch 4 times per week<br>• Tips and inspiration for parents on making healthy midmorning snacks and possibly lunch<br>• 3x40 minutes of organized vigorous PA per week in school.<br>• PA in breaks and after school clubs.<br>• Inspiration sheets for families on screen media habits, sleep and dietary habits etc.<br>• Tips and inspiration for staff in after-school clubs on how to follow the national food-based guidelines<br>• Cold-water dispensers, if relevant<br>• Parent workshops at the school<br>• Workshops, focus groups and interviews with children<br>• Assignments / exercises for children during school time about screen media habits, sleep, nutrition and food competences<br>• In the local community surrounding eight intervention schools (six municipalities): Workshops with local stakeholders and establishment of local community partnership groups.<br><br>**Other**<br><br>• School staff education<br>• Packages of loose play and sports equipment for school classes<br>• Community events<br>• Fieldwork, interviews, focus groups, logbooks, and short questionnaires for e.g., children, parents, school staff and local stakeholders<br>• Observations

6.6 Measurements and study procedures

As mentioned, measurements will be conducted in 3 test rounds i.e., at the beginning of the first school year and at the end of each of the two school years. Each measurement will be conducted in all 3 rounds, unless stated otherwise below.

6.6.1 Questionnaires to be filled in at home

The questionnaires will be administered via text message and e-mail with a link to the electronic survey to parents. All questionnaires will be offered in Danish, but participants can choose an English version for some of the questionnaires.

The following questionnaires will be administered:

- Family background (round 1 or at round 2, 3 or at new enrolments if not answered at previous data collection rounds): Data on children's SES will be collected from the parent(s) or legal guardian(s) using the Danish Occupational Social Class Measurement (93) (appendix 10).
- Sun habits (vitamin D) (in appendix 10)
- Child's leisure time sports participation (in appendix 10).
- Pubertal stage (round 1 and 3) (appendix 11-12).
- Food allergies (only intervention schools), plaster allergies, and chronic disease (Appendix 2A for intervention schools and 2B for control schools)
- Neurodevelopmental disorders and medical conditions (in appendix 2A and 2B) (at round 1 or at round 2, 3 or at new enrolments if not answered at previous data collection rounds).
- Breakfast consumption, intake of beverages, fish, and dietary supplements (appendix 13).
- Wellbeing & Quality of Life: Children's well-being will be measured by Strengths and Difficulties Questionnaire (SDQ) (appendix 26). As no valid measure exists for children under age 11, this parental questionnaire will be used to supplement child-reported well-being.
- Child and parent screen time, type and current screen time practices will be collected using the modified parent reported SCREENS questionnaire (95) (appendix 15).
- Child quality of sleep will be measured using the Children's Sleep Habits Questionnaire translated to Danish (96) (appendix 16).
- Family grocery shopping habits (round 1 and 3, only for parents in the six intervention communities where the expanded local community intervention takes place) (appendix 25)

- Perception of the project and the activities (see section 6.6.4).
- Adverse events and concomitant medicine (round 2 and 3) (appendix 17).
- Drop-out questionnaire (at dropout) (appendix 3).

6.6.2 Other procedures at home

6.6.2.1 Dietary records

Children's daily intake of food and drinks will be recorded by the parents for three consecutive days (two weekdays and one weekend day). We will use the validated, web-based tool, myfood24, which uses data from the national food composition tables. The tool is user-friendly, suitable for the target group and recording links can be sent via email.

6.6.2.2 Screen time and categorized app time measurement

At baseline, we kindly ask the parents to install a research-based app, Ethica (https://ethicadata.com), on the participating child´s *own* smartphone and/or tablet. At all test rounds, we will use Ethica to collect information on screen time, categorized app usage and screen time patterns over a period of 14 days. After installation, the app runs automatically in the background. The app has been developed to meet legal requirements for data security.

6.6.2.3 Physical activity and sleep assessments (Axivity)

Detailed, objective information on the whole spectrum of behaviours will be assessed by continuous 24-hour/7-8-day Axivity® accelerometer measurement using thigh positioned monitors. Using the thigh measurement position the modified age-adjusted method of Skotte et al. (97) provides the ability to classify postural allocations like sitting, standing and activities like walking and running with high sensitivity and specificity in children (98). Time lying in bed and sleep time will be objectively measured using an accelerometer classification algorithm that is currently under development.

The Axivity® AX3 monitor is a small (23 x 32.5 x 7.6 mm), lightweight (11 g), waterproof, 3-axis accelerometer data logger (Axivity, 2016). The Axivity® AX3 will be attached to the skin using thigh belts developed by a Danish company (Elas) or tape (Opsite Flexifix®, Fixumull®) directly on the body using compress (gaze) or artificial skin (DuoDerm) between the monitor and the skin. We have good experience with both methods attaching the Axivity® AX3 monitor to the body, but using plaster is more time consuming so we need to find out which method is most feasible in the study. The monitors will be attached at the school. All observers/staff in contact with the children will undergo pedagogical and professional training in the tests and in carrying

out the measurements with the children and in adherence with Danish law have the necessary criminal records bureau check. It is important to ensure that the children also give their assent to participate in the study, if their parents have provided a written consent allowing them to participate. During the study period we will be aware of the children´s reactions and whether they feel comfortable or not with the test situations. If the children do not want to wear accelerometers, we will respect this. Children with plaster allergy, childhood eczema or sensitive skin will always be offered to use belts. The children or parents will receive an information leaflet to take home or on the parent email including relevant information regarding the physical activity measurement.

6.6.3 Measurements in school

Two program centres (UCPH in DK-East and SDU in DK-West), with specially trained test teams, will be responsible for the measurements in the schools. The measurements in school will be spread over 2-4 days for each child.

6.6.3.1 Measures of anthropometry including height, weight, and waist circumference

The children will be weighed once in underwear/light clothes on a digital scale. Standing height will be measured three times to the nearest millimetre with a stadiometer. Waist circumference will be measured three times to the nearest millimetre by a non-elastic measuring tape at the level of the umbilicus. The mean of three measurements will be used for height and waist circumference.

6.6.3.2 Body composition and blood pressure

Body composition (i.e., FM and fat free mass) will be measured by air-displacement plethysmography using a BOD POD and a bioimpedance weight (InBody 270). The children will be wearing underwear/light clothes and a bathing cap. The children must sit in a transparent capsule for approximately 5 minutes (BOD POD). The bioimpedance measurement takes approximately 1 min.

Blood pressure and resting heart rate will be measured three times by an automated device while the child is lying down after 10 minutes of rest. The appropriate cuff size for the child's arm circumference will be used.

6.6.3.3 Blood samples

Blood samples will only be collected from children in DK-East (12 schools). This is due to logistic and budgetary reasons, and furthermore, power calculations show that blood sampling from children in 12 schools will provide us with sufficient statistical power for the study outcomes

that are measured in blood samples (see power calculation in section 6.1.4). Therefore, we consider it as an important ethical consideration that blood samples are only obtained from the statistically sufficient number of children. All parents and children will follow the same informed consent procedure in accordance with the Committee Act, regardless of whether blood samples are obtained (see sections 6.2.5.1-6.2.5.6).

Blood samples will be collected in the overnight fasted state (except water) only from children in DK-East. The children can have breakfast shortly after the blood samples have been collected. We will instruct the parents to provide the children with a breakfast pack on the blood sampling day but will also have food available for children that do not have any. The children will be provided with local anaesthetic patches (EMLA), which the parents will be instructed to apply on their child's arms in the morning (appendix 18). After removal of the patches, max. 30 ml blood will be drawn from the child's forearm by trained laboratory technicians. The blood samples will be brought back to University of Copenhagen for processing and storage at –70°C until analysis.

6.6.3.4 Health related fitness and motor functions

Aerobic fitness and intermittent exercise performance will be evaluated by the Yo-Yo Intermittent Recovery Level 1 Children's test (80), where the children will have to run 2x16m at increasing speed with 10 s break between each run. The test continues until the children cannot reach the finish line in time and takes around 20 min. for up to 50 pupils, depending on the size of the indoor gym. Heart rate will be measured continuously during the test, to determine endurance capacity and maximal heart rate. The test is simple and validated for fitness and maximal heart rate assessment for 6-10-year-old children (99). Heart rate and locomotor activities will also be measured during selected physical activities throughout the study period to describe the intensity.

Muscular fitness, strength and sprint performance will be determined by the hand grip strength test and a 20-m sprint test. The hand grip strength test is conducted with a hand grip dynamometer. The child squeezes the dynamometer with all their strength, two times with each hand. An average score is then calculated using the measurements from both hands. Sprint time is measured by photocells. The children will complete each test twice and will be instructed to use all their power.

Gross motor functions are assessed as static and dynamic balance ability, upper extremity gross-motor function, jump height and agility. Jump height and balance ability are measured on a force plate measuring the vertical ground reaction force. Jump height is measured during the vertical countermovement jump test. Balance ability is measured during one-leg maintained balance and as maximum reaching ability during maintained balance, Agility is

measured as time to complete an agility running exercise with different motoric tasks. Time to completion is measured by photocells. The children will complete each test twice and will be instructed to use all their power. Upper extremity gross-motor function is tested as speed and accuracy in the ability to perform accurate, goal-directed reaching movements towards a number of visually displayed targets within 30s or 1 minute.

6.6.3.5 Test of cognitive functions and school performance

The children will complete a short battery of standardized, validated and age-appropriate neurocognitive tests (100,101) including tests of executive function, memory, and attention. The tests resemble computer games, and they are administered individually via a tablet or iPad. In addition, school performance will be assessed by use of standard and age-appropriate tests of Danish spelling, reading and mathematics proficiency (100,102). We have good experience administering these methods in a school setting and for children at this age.

6.6.3.6 Wellbeing & Quality of Life: KIDSCREEN 27

Children's well-being will be measured using KIDSCREEN 27 (appendix 14). This validated questionnaire consists of 27 items that measure five dimensions of children's quality of life and well-being. These are physical well-being, psychological well-being, relationship with parents, relations to peers & social support, and life quality in the school environment (94). Due to the respondents' low age and inability to read, a video and speech assisted child version of these questionnaires will be developed. The children will fill in this questionnaire using tablets and headphones enabling all items of the questionnaire to be read aloud, while the text appears on the screen. The answer scale points are illustrated with smileys.

To assess pupils' psychological well-being and functioning during school-based intervention activities, basic psychological needs satisfaction and intrinsic motivation is measured acutely right after occurrences of the school intervention activities. Satisfaction of children's three basic psychological needs for experiencing relatedness; - competence and autonomy as well as intrinsic motivation - will be measured with 3 items each adapted from Basic Psychological Needs Scale (BPNS-R) for PE (103) and the Intrinsic Motivation Inventory (104,105) adapted to the age-group and context (Appendix 18). A smiley based 4-point answer scale will be used.

To assess the motivational climate during intervention activities, systematic observations and scoring of indictors of empowering (task-orientation, relatedness support and autonomy support) and dis-empowering climate (ego-orientation and controlling behaviour) will be collected during intervention activities.

6.6.3.7 Motivation, confidence, and knowledge in relation to physical activity

A Danish video-assisted questionnaire, MyPL questionnaire, validated for children down to 7 years of age will be used to measure children's intrinsic motivation and confidence for physical activity as well as their knowledge and understanding of physical activity. The MyPL is a 20-item physical literacy assessment tool that strives to account for how the domains of physical literacy (motivation, confidence, physical competence, and knowledge in relation to physical activity) differs across different social and physical environments for physical activity, as described in the conceptualization of physical literacy by Whitehead (113). This structure makes it cognitively easier for the children to think about and thus answer. It consists of five subscales reflecting context and discipline-specific motivation, confidence, and knowledge for physical activity. The children will fill in this questionnaire using tablets and headphones enabling all items of the questionnaire to be read aloud, while the text appears on the screen and introduction supported by pictures (Appendix 19).

The children will also be asked about their participation in leisure time sports club activities (Appendix 20).

6.6.3.8 Food competences and meal culture

Children's food competence and meal culture will be assessed using a self-constructed questionnaire developed for and validated in the age group (Appendix 21).

6.6.3.9 Registration of snack and lunch consumption

On selected days, portion sizes and food/beverage intake at breakfast, midmorning snack, lunch and afternoon snack in schools/after school clubs will be recorded at class level in a subsample of the schools. Also, teachers/pedagogical staff present in the classes during the children's consumption of lunch will be asked to roughly assess and register the intake of this meal at class level.

6.6.4 Evaluation of measures related to implementation of the intervention and organizational aspects

To explore acceptability, perceived outcomes, and consequences of the intervention, as well as structural and organizational aspects related to implementation of the intervention, school management at each school, and/or other relevant personnel with coordinating/managerial responsibilities related to this project, will be asked to complete questionnaires and to participate in interviews. Moreover, all teachers and/or other relevant pedagogical staff, affiliated with the various intervention components, will be asked to complete surveys and to

participate in interviews. Interviews, focus groups and brief surveys (Appendix 23 and 24) will be conducted with children in order to investigate the children's perceptions of the Generation Healthy Kids program components, as well as how the intervention influences children's perceptions of concepts such as health, body and well-being. Field work during school hours, e.g., classes, PA sessions, recess, and lunch with focus on how the intervention unfolds in practice, reasons for adaptation, and participant responsiveness will be conducted via observation methods etc. In addition, we will conduct process evaluation fieldwork (observations and on the spot interviews) in relation to measurement days at baseline to explore how the children respond to and experience the measurements.

Parents will be asked to participate in interviews and to complete questionnaires regarding perception of the project and the activities. Moreover, PE teachers are asked to roughly assess and register attendance and active participation during the 3x40 minutes sessions of PA at class level. Local coordinators will be asked to register the number of parents who attend school events, to explore implementation and perceived outcomes of the intervention. Moreover, fieldwork will be conducted when possible.

To explore relevant structural and organizational aspects as well as initiatives focusing on diet, physical activity, screen time and/or sleep in the control schools, the school management at each control school will be asked to complete a (few) questionnaire(s) and participate in an interview.

6.6.5 Measurements at further school, community, and national level

During the study, we will track the implementation and stakeholder engagement. Implementation data will include a register of activities in each local community, a database of stakeholder engagement and key engagement activities. Data will be collected on a regular basis and use methods recently described by Maitland et al. (107).

Furthermore, we may collect structural data on food environments, including accessibility and spatial layout of stores, using digital maps, GIS data (e.g., Smiley register), field studies and sales data from supermarkets (90).

We plan to conduct both a) interviews with the manager of the after-school club and facilitators of activities in the leisure time clubs and the active summer holiday camp as well as b) systematic observations on selected days to explore acceptability, perceived outcomes and consequences of these intervention components. Moreover, we expect that actors involved in the implementation of intervention activities, e.g., schoolboard members, managers of after-school club, volunteers in leisure time clubs, will be asked to fill out questionnaires and/or take part in interviews exploring structural and organizational factors

influencing implementation. Several core theories, models and frameworks within implementation science will serve as reference points for the mentioned investigations.

We will measure local and national partnerships using the Danish translation of the VicHealth partnerships analysis tool (108). Furthermore, this tool will be adjusted and adapted to the current project inspired by the work done by Indig et al. (109).

6.6.6 Use of the school's recordings of children's absence

As an indicator-measure of intervention effects on the children's general health and school wellbeing, we will seek to obtain the schools' recordings of children's attendance and absence, by parental consent.

6.7 Adverse Events

Adverse events (AEs) will be registered during the intervention. At each round of measurements, parents/custody holders will be asked by questionnaire if their child has had any AEs or reactions and any new medicine or changes in dosage taken since the last measurement as well as the start and end date (Appendix 17). In addition, parents will be asked to contact us if any events or reactions occur. The medically responsible physician will evaluate and record the intensity, causality, actions needed and outcomes of reported events.

6.8 Handling of biological samples

6.8.1 Sample analysis

Biological samples will mainly be analysed at University of Copenhagen, Denmark. Samples could also be analysed at other laboratories within and outside the EU, when this is judged necessary for the quality of the analyses. Therefore, it is not possible for researchers to predict in advance where analyses will be carried out and by whom. If data is transferred to countries outside EU to a country which cannot ensure an adequate level of protection, the transfer will be in accordance with chapter V of the GDPR. Parents will be informed that their data may be sent to data processors in a country like these if this is necessary for the quality of the overall result of the study. They are also informed that their data will be sent as pseudonymised/coded data and that the transfer will comply with the GDPR.

Parents will be informed separately about the handling of the data and all data transferring will be in accordance with GDPR.

The processing of data and biological samples in the project and at external data processors will be recorded at the mandatory lists to the Danish Data Protection Agency, and all data processing will follow GDPR/Databeskyttelsesforordningen and Databeskyttelsesloven. Data set and samples will always only be sent to data processors in coded s without any accompanying data that could potentially identify the participants.

Table 2 gives an overview of the planned analyses of the biological samples.

Table 2. Biological sample analyses – blood

Biomarker field	Analyses
**Compliance & nutrition biomarkers**	• Fatty acids, lipids and other nutrient biomarkers<br>• Alkylresorcinols (wholegrains) and other food biomarkers<br>• 25-hydroxyvitamin D, vitamin D binding proteins and -metabolites<br>• Haemoglobin, ferritin, and transferrin receptor (iron)
**Growth & development**	• IGF-I, IGFBP-3 and other growth factors<br>• Osteocalcin, bone specific alkaline phosphatase and other bone markers<br>• Parathyroid hormone<br>• Sex hormones
**Cardiometabolic**	• Triacylglycerol and total, LDL and HDL cholesterol and others<br>• Glucose, insulin, C-peptide, glycosylated hemoglobin (HbA1c) etc.
**Appetite**	• Appetite hormones
**Inflammation**	• C-reactive protein, cytokines, adipokines, and related markers<br>• Immune cells and immune markers
**Cognition**	• Blood markers related to brain function such as brain-derived neurotrophic factor (BDNF) and serotonin
**Mechanisms**	• Metabolomics, proteomics and lipidomics<br>• Short chain fatty acids and other metabolites

**Genetics**	• Genotypes and epigenetics related to the study outcomes and research questions (single nucleotides polymorphisms and genome wide association studies, NOT full genome sequencing, and epigenetics)

6.8.2 Genotypes and epigenetic analyses

Gene polymorhisms and epigenetic analyses will be used to investigate biological mechanisms and effect modifications, as described in section 5.2.2. We will only investigate genes related to the exposure variables and study outcomes, e.g. involved in nutrient status, growth and body weight, metabolism, cognitive function etc. Genotyping will not include any full genome or exome sequencing, but will either be performed by DNA microarray (such as the Illumina Infinium HumanCoreExome Beadchip), that gives information on common gene variants (>5%), or by targeted analysis of common single nucleotide polymorphisms. It is therefore considered highly unlikely that we will identify any genetic variants that have any direct relevance for illnesses.

6.8.3 Research Biobank

In relation to the planned analyses in this study, a research biobank will be created at Department of Nutrition, Exercise and Sports, University of Copenhagen for storage of blood samples. The research biobank will include maximum 3 x 30 mL = 90 mL of blood from each child.

The research biobank is registered at University of Copenhagen. Biological material will be stored at maximum -70 C in the research biobank until analysis is completed and will be destroyed no later than 10 years after last participant last visit (June 2035), unless custody holders have signed a separate consent form that eventual remains can be transferred to a biobank (see 6.7.3).

All personal data/biological material is handled with confidentiality and stored in accordance with applicable law, GDPR and Danish Data Protection Act. no. 502 of 23rd May 2018.

6.8.4 Biobank for future research purposes

Eventual remains of material of blood (maximum 3x 20 mL=60 mL) from the research biobank, will, if the custody holders of participants have signed a separate consent form for this, be

transferred from the Research Biobank to a biobank at University of Copenhagen. The biobank material will be stored at maximum -70 C at the Department of Nutrition, Exercise and Sports, University of Copenhagen, Rolighedsvej 26, 1958 Frederiksberg C. The biological samples in this biobank will be stored for a maximum of 20 years after last participant last visit (June 2045) and will only be used in new research projects after these have been approved by the Danish Regional Committee on Biomedical Research Ethics, unless the samples have been totally anonymized, in which case they will be stored longer. Anonymization means that the ID-log that connects study participants and the ID on the biological material has been destroyed and it is no longer possible to identify the participant. If the study participants or custody holders of participants choose to donate remains of biological material from the research biobank to the biobank and later regret it, they can contact the Department and have the samples destroyed if the samples have not been anonymized.

The PI knows that establishment of a biobank for future related research projects of the excess material from the research biobank is not covered by the committee by law, but by the General Data Protection Regulation (Databeskyttelsesloven/ Databeskyttelsesforordningen). As the procedure is considered relevant for the entire research protocol in Denmark and to be able to prove that the biobank is established on a legal basis, the information is included in the material for the ethical committee.

6.9 Data Management and Quality Control

6.9.1 Data Collection and Processing

All collected data will be stored in accordance with the General Data Protection Regulation/Databeskyttelsesforordningen for data handling.

6.9.1.1 Data storage in REDCap

Data from questionnaires and most outcome data collected during school measurement days will be collected and stored directly in REDCap, which is a secure platform for building and managing online surveys and databases. REDCap is managed by the researcher service organization OPEN in the Region of Southern Denmark.

Research personnel will collect and enter data following formulated standard operating procedures. Data quality will be ensured using tailored validation fields in REDCap.

6.9.1.2 Data storage on other platforms

Outcome data (smartphone use, cognitive tests and dietary record) will also be stored on online servers provided by external companies (Ethicadata, Cambridge Cognition, MyFood24). We will establish data handling agreements with the companies to ensure that data is securely and not used for other purposes.

Data from heart rate monitors (collected during the fitness test) will be extracted and stored pseudonymised in safe folders on university servers at SDU or UCPH.

Accelerometers will be collected at the schools (after 7-10 days) and transported back to the University of Southern Denmark or University of Copenhagen where data will be extracted from the measurement devices and stored in safe folders on the University servers.

Collected blood samples will each day be transported back to the University of Southern Denmark or University of Copenhagen where they will be processed and stored at maximum –70 degrees Celsius until analysis.

Process data such as field notes, observations, logbooks, and interviews, which will be collected in paper form or recorded, will be entered and stored in safe folders on university and/or region servers at SDU and/or Center for Clinical Research and Prevention.

Aggregated datasets from all data storage platforms other than REDCap will be stored in the REDCap file repository. The aggregated datasets will be uploaded to the REDCap file repository and include the ID log variable to allow for combination of the datasets.

6.9.2 Confidentiality

To maintain confidentiality, participants will only be identified by their initials and an assigned participant number. Documents that identify the participant (such as the signed informed consent document and the ID log) will be maintained in strict confidence by the investigator, except to the extent necessary to allow auditing and/or monitoring by the appropriate regulatory authority. All information obtained during the study will be handled according to local regulations and the GDPR/Databeskyttelsesforordningen. The study is registered on a central list at the Institutions/UCPH and SDU. The universities conduct lists of all data controllers/data processors and their handling of the personal data, and the lists are available in case of inspections from the Danish Data Protection Agency.

6.9.3 Information/data from patient hospital records

Data and information from the participants' Hospital medical records will not be included in this project.

6.10 Use of data from Danish registers

We will obtain information on eligible children and their families from Danish registries with the purposes to 1) obtain contact information (name, address, and CPR-number) on eligible children and their parents (for distribution of all participant materials related to the study), 2) compare socio-demographic characteristics (parent education and employment) of participants with eligible non-participants to better judge generalizability and explore this analytically, 3) investigate the long-term (i.e., a 7-year follow-up) effectiveness of the program on body composition, and 4) lower the burden of the parental survey with respect to sociodemographic information, and 5) limit missing information on sociodemographic information due to survey non-response. Registries that will be used for these purposes include at least the following: The Civil Registration System (Det Centrale Personregister), The National Child Health Register (Børnedatabasen), and the DREAM database.

In the participant information (Appendix 5A and 5B), we inform all parents that we plan to collect information on the above-mentioned factors from Danish registers on all eligible children regardless of whether consent to participation in this project has been obtained or not. The Legal authority for said collection of data will be Section 10 in Databeskyttelsesloven and Article 6 (1) (e) of General Data Protection Regulation

Furthermore, we will obtain information from Danish national health registers and clinical databases, e.g., the National Patient Register, the National Prescription Register, the National Health Services Register, the Danish Psychiatric Central Register, the Medical Birth Register, and the National Child Health Register, to study associations between lifestyle factors and biomarkers collected in the study and subsequent long-term health and morbidity among participants. Such registry linkages will be performed for up to 10 years after last participant last visit (June 2035) to be able to passively follow the cohort during adolescence and adulthood, and thereby study early childhood determinants of later health and morbidity. All registry linkages will be performed on secure servers at Statistics Denmark or the National Board of Health Data, and all data sets will be pseudonymized before analysis.

The establishment of a database containing data from this project as well as the subsequent transfer of data for related future research projects are not covered by the Danish Committee Act. Rather, for said actions, PI must comply with the General Data Protection Regulation (Databeskyttelsesforordningen) and Databeskyttelsesloven. Accordingly, the parents must be

informed separately about this in the mandatory GDPR information sheet and a valid consent must be obtained.

6.11 Statistical methods

Reporting of the trial will be done in accordance with the CONSORT guidelines for cluster randomized trials.

6.11.1 Primary analyses

The analyses of the predefined primary outcomes will be planned a priori and made publicly available in a statistical analysis plan at clinicaltrials.org. The primary analysis will be conducted on an intention-to-treat basis using mixed regression analysis with school as random effect using degrees-of-freedom correction (Satterthwaite) to control for type 1 error due to the moderate number of clusters. Because the school is the unit of randomization (not municipality, region, or school-class) and the intervention is being applied at this level, no further cluster-level will need to be considered statistically. Analyses will be adjusted for baseline factors that are found to be related to individual schools (clusters) to control for possible baseline imbalance of cluster- and participant level data. In analyses using more than two assessment points, random slope effects of time will be considered to examine the possible heterogeneity of effects according follow-up time. In all intention-to-treat analyses, data from all participating children at inclusion will be included. All hypotheses testing will be based on two-sided tests at the alpha=0.05 level.

In addition, several sub-group analyses will be predefined and outlined in the final statistical analysis plan before the start of the trial. This will include subgroup analyses by e.g., parent socio-economic status, school district characteristics, and baseline adiposity status (being normal weight at baseline vs. being overweight or obese at baseline). Sub-group analyses among normal weight children at baseline will also be used to examine the effectiveness of the program on incidence of overweight or obesity. Other outcomes or sub-group analyses that are not predefined in the final study statistical analyses plan published before the start of the trial, will be defined explicitly as exploratory post hoc analyses in the papers and the exploratory nature of these analyses will be mentioned as a major limitation. Per-protocol and complete case analyses of the primary and secondary outcomes will also be carried out. We will analyze the association between implementation fidelity and actual dose delivered of each intervention activity in each intervention setting and its association with intervention outcomes among children.

In the final statistical analysis plan, a detailed plan to adjust for multiplicity based on a priority of the secondary outcomes and follow-up time points will be outlined.

6.11.2 Secondary analyses

Analysis and reporting of predefined secondary outcomes will be mentioned explicitly as secondary outcome analyses in the papers.

6.11.3 Exploratory analyses

All analyses that were not pre-planned will be labelled as exploratory analyses.

6.11.4 Safety analysis

A safety analysis will be carried out to summarize any reported adverse events associated with participation in the study.

6.11.5 Definition of datasets

Quantitative datasets will be generated from the REDCap databases for each specific research aim guided by a predefined analysis plan. When registry data are needed for analyses, and these cannot be stored locally in REDCap, data stored in REDCap will be uploaded to 'Forskermaskinen' at Statistics Denmark.

6.11.6 Handling of missing values

Missing values will be handled via the mixed regression modelling approach to analyses (110,111). If alternative analyses are carried out missing values will be imputed using multiple imputation or inverse-probability weighting.

6.12 Suspension or early termination of the study

The principal investigator may suspend or prematurely terminate parts of or the entire clinical study for significant and documented reasons. If a suspicion relating to an unacceptable risk to study participants arises during the study, the principal investigator will suspend the study while the risk is assessed. The principal investigator will terminate the study if an unacceptable risk is confirmed. The principal investigator must ensure that the premature termination is justified in writing (fill out the termination form) and that the parents of the participants are informed.

6.13 Serious adverse events and reporting

6.13.1 Serious adverse events

A serious adverse event is defined as any untoward medical occurrence in a study participant that:

- Results in death
- Is life-threatening at the time of the event
- Requires inpatient hospitalization
- Results in persistent or significant disability or incapacity.

6.13.2 Reporting of Serious adverse events

The principal investigator will immediately inform the ethics committees if a serious adverse event occurs during the project. Reporting will take place no later than 7 days after the principal investigator became aware of any such adverse event. The standard forms from the ethics committees will be used for this purpose. In case of serious adverse events resulting from the project, the investigator will make any information requested by the committee available.

When the study ends, the principal investigator will provide a list of all serious expected and unexpected adverse reactions and all serious adverse events that have occurred during the study period. The report will be accompanied by an assessment of the participants' safety. The report will be delivered using the forms prepared by the committee system.

6.14 Study Documentation

6.14.1 Publication of Results

Results from the study, whether positive, negative, or inconclusive, will be summarized and published in peer-reviewed scientific journals and/or as part of scientific conferences. Authorship of publications should in agreement with the publication approval procedure reflect the collaborative nature of the project.

The project is registered in www.clinicaltrials.gov

7 Ethical conduct of the study

The study will be conducted in accordance with the ethical principles set forth in the current version of the Declaration of Helsinki. Children in the participation classes that are not enrolled in the study will be invited to participate in all activities that are like what could happen during the school day anyway, but without any examinations and data registration.

It always, and especially for families with small and younger children, interferes with everyday life to participate in a clinical study as it requires time to be set aside to comply with the procedures of the study, and some procedures may be associated with a risk.

7.1 Ethical approval and study registration

Ethical approval is obtained by the Scientific Ethics Committee in the Region of Southern Denmark (Project ID S-20220094) and the data processing of the participants' personal data and biological material and the study is registered on statutory lists before enrolment of any study participants.

7.2 Justification of the study

Investigation of the effectiveness of a health promotion intervention program in children on health outcomes is not possible to conduct with high validity in a non-randomized experiment or in an observational study. Thus, for the present study there is a need for a control group and to investigate the aims of the study using an experimental randomized study design. The study will provide important new knowledge about the effectiveness of the Generation Healthy Kids program, a novel multicomponent intervention developed using a systems approach that can benefit children in Denmark and elsewhere in the future.

7.3 Underage study participants - justification and precautions

Justification of inclusion of children in the clinical study is in line with the Committee act (Komitéloven §19, stk. 3) which concerns studies not involving medicine, as the project can only be conducted in the described age group to answer the objective stated in section 5. Further, it is justified to recruit children of this young age as the overall project and main trial is focused on early prevention of overweight at school start before the overweight prevalence starts to rise markedly. Also, as described below, the study gives minimal risk of harm for the participants, and it is our assessment that the benefits outweigh the potential harms.

7.4 Children of ethnic minority

All children in the participating classes are eligible for the study. Written information materials will be translated depending on what is needed in the local school district. Verbal information will as standard be in Danish, but interpreters will be present at meetings if needed.

7.5 Risks and safety in relation to conducting the study

The interventions will not include focus on weight, but instead promote well-being, healthy habits, sense of community, and play. All communication will be prepared carefully in terms of non-stigmatization of children or families and there will not be any focus on weight related issues.

Participation in the study is associated with minimal risks. We have thoroughly considered the ethical aspects of the discomfort of the biological sampling (only children in DK-East). Since the blood samples are crucial for investigating the outcomes of the study (including diet and health related biomarkers), the discomfort of the blood sample is considered to be outweighed by its usefulness. The discomfort of blood sampling will be minimized by use of local anaesthetic patches before blood sampling and blood will be sampled by skilled laboratory technicians or other trained and qualified staff. The only expected risk of the biological sampling is a mild pain at the site of the blood sampling on the arm and potentially a small blue bruise at the site of the prick, which will disappear within a few days. Risks for infections that are associated with blood sampling are extremely low.

The children in DK-East will be asked to meet for the visits after an overnight fast to standardize the blood results. They have time to eat immediately after the blood sample, at 10:30 am the latest, and, as in our previous trials with blood sampling in children they are allowed to drink 100-200 ml during the fasting period and up to 1-2 hours before the blood sampling. A fasting period of this duration is relatively short, and we do not expect the children to experience considerable discomfort due to the fasting. However, if this should be the case, the examinations will be discontinued immediately, and the child will be offered drinks and food.

The BOD POD body composition measurement is crucial for investigating the primary outcome of the main trial (fat mass). The measurement uses air-displacement plethysmography, is safe and approved for children, and takes few minutes. The measurement is constantly monitored by the operator and if the child expresses discomfort sitting inside the BOD POD, the measurement will be stopped immediately. Bioimpedance measurements are also non-invasive and not associated with risk or harm.

There is a risk of more focus on weight related issues e.g., overweight when measuring height, weight, and waist circumference. Therefore, the personnel will be trained to talk carefully about the measurement and to handle comments on body weight related issues from peers. Moreover, the children will not be told the measurement numbers (numbers on digital scale is hidden) and they can wear light clothing during the examinations.

There are no considerable risks related to the school meals which are prepared using foods that are part of a normal diet. The parents will be asked about food allergies at project start-up and will be informed to check the menu plans with ingredients allergens online the week before. The children will be instructed in hygiene e.g., hand washing. The risk of injury or discomfort of performing the muscular and fitness tests are comparable to the risk children are exposed to during daily life and play. In case of any discomfort or accident the activity will be discontinued.

The risk or side effect of participating in the measurement of physical activity based on our extensive experience in carrying out physical activity measurement in paediatric populations is minimal. However, attaching the monitor to the skin on the thigh, might be unpleasant for some children and might in some rare cases result in skin irritation or an allergic reaction to the adhesive tape. The parents and the child will receive written and oral instruction about the instruments, and how the instrument can be removed gently. Furthermore, the written information will contain information on which symptoms to be aware of to reduce any physical inconveniences in case of an allergic reaction, and whom to contact in case of an allergic reaction. If the children participating in this project are allergic to adhesive tape, have eczema or other skin problems (see appendix 2A and 2B), they will be offered to wear a monitor in a thigh belt. The belts include skin friendly silicone to keep the belt in place during the child´s play. Children/parents can switch the belt between the right and left leg if necessary and to avoid/reduce potential skin irritation.

7.6 Benefits of the study and risk-benefit evaluation

The overall aim of Generation Healthy Kids is to promote healthy weight and wellbeing in children and is expected to benefit future generations of children. Furthermore, it is expected that the study will generate new and important knowledge on how to promote sustainable changes and potentially how to scale-up.

The participation in a study can cause some inconvenience to daily life and can be time-consuming. During the study, the families in the intervention schools will receive free school meals for the children, inspiration, and support for healthier lifestyle habits, and at the control

schools the participating classes will be invited to an activity day by the end of the study period and teachers will be offered education in FIT FIRST teen. All participating children will receive a small gift at each measurement round (see section 9), and after completion of the study all children will receive a diploma (Appendix 22). After data analysis, and publication of the main scientific papers, the parents can read about the study's main conclusions on the project's website. In addition, the parents of children that have provided a blood sample will receive the child's iron status. The schools and local communities are also expected to benefit from the study in terms of elevated knowledge and focus on a healthy lifestyle.

All things considered, the study is judged to be ethically justified and proper as the individual, scientific and societal benefits are believed to outweigh the limited discomfort and very low risk of harm associated with the study.

7.7 Participant Information and Informed Consent

Written, informed consent will be obtained from all parents/legal guardians to the subjects prior to entry into the study and execution of any study-related procedures. PI or delegated and qualified project staff will explain verbally to parents/legal guardians the objectives, nature, significance, risks, and implications of the study before inclusion. This information will also be included in the written subject information sheet. The information will be given at meetings at each school. If the parents/legal guardians are not able to join the meeting, they can arrange a separate meeting with project staff.

The parents/legal guardians will be offered to bring with them an assessor to the information meeting and after the meeting they are given time to discuss any questions and decide on participation in the study. All parents/legal guardians will receive a copy of the subject information sheet. The children will be informed of the objectives and procedures of tests in a language they can understand. The procedures are explained in more detail in section 6.2.5 and in the participant information.

7.8 Protocol Changes

Substantial amendments to this protocol may be implemented only after a favourable opinion of the Ethics Committees has been obtained. Amendments to the protocol are regarded as substantial if they have a significant impact on:

- The safety, physical health, and mental integrity of the study subjects
- The scientific value of the study
- The conduct or the management of the study

7.9 Protocol Deviations

No systematic deviations from the protocol are allowed. All significant protocol deviations noted during the study (by the Investigator) will be recorded in the e-CRF (REDCap).

7.9 Insurance

The participants are insured in accordance with the Law: "Lov om arbejdsskadeforsikring" (cf. LBK nr 1186 af 19/08/2022) by the current insurance at University of Southern Denmark and University of Copenhagen. During the study the participants will be covered by law: "Lov om klage- og erstatningsadgang inden for sundhedsvæsenet" (cf. LBK no. 995 14/06/2018 www.retsinformation.dk).

8 Funding

The initial concept of the Generation Healthy Kids was developed by the Investigators of the study. Generation Healthy Kids is funded by the Novo Nordisk Foundation with 80 mio. DKK in total, covering both the pilot study and the main trial. The study funds have been transferred to a research account at University of Copenhagen, which is subject to public audit. The funding will primarily cover salary for project staff (PhD students, postdocs, medical laboratory technicians, project managers and communication staff) and expenses related to operation e.g., school food, buy out of teachers, measurement equipment, analysis of blood samples, car rent, and communication materials. Funding will not be used for paying subjects.

The Investigators declare to have no financial interests in the results of the study or any economic affiliation with any of the project partners.

9 Remuneration for study participants

There will be no remuneration for the study participants. In addition to the free school meals, which will be offered to all children in the intervention school classes, all participating children will receive a small gift (e.g. a sticker, LEGO figure or key ring) at a value of up to 30 DKK at each measurement round. Also, due to the inconvenience of the blood samples and the preceding overnight fasting, children whose parents have consented to blood sampling in DK-East, will receive a small gift (e.g. a small LEGO package) at a value of 70-130 DKK at each round of blood sampling. Parts of the gifts have been donated by LEGO Charity and the gifts are similar to those provided in Danish hospitals, when children have blood samples taken.

Considering this, and the small size of the gifts, it is our assessment that it will not affect the willingness to consent and is therefore ethically appropriate.

10 References

1. WHO. Report of the Commission on Ending Childhood Obesity. 2016.

2. WHO. Global status report on noncommunicable disease 2014. Geneva: WHO; 2015.

3. Danmarks Statistik (DST) og Danmarks Tekniske Universitet F (DTU). Ulighed i børneovervægt i Danmark. 2021.

4. Marie Borring Andersen, Anne Thorsted, Andrea Huber Jezek, Pernille Due, Thorkild I. A. Sørensen, Lau Caspar Thygesen. Overvægt og svær overvægt blandt danske børn og unge. 2020.

5. Jensen H, Davidsen M, Ekholm O CA. DANSKERNES SUNDHED – Den Nationale Sundhedsprofil 2017. 2018.

6. Dennison N, Sollars L, Arrigon D, Pridgeon A. Promoting healthy weight in children, young people and families: A resource to support local authorities. 2018.

7. Pant SW PT. Sundhedsprofil for børn og unge ind- og udskolingsundersøgt i skoleåret 2018/19. Databasen Børns Sundhed. København; 2020.

8. Danmarks Statistik (DST) og Danmarks Tekniske Universitet F (DTU). Ulighed i børneovervægt i Danmark. 2021.

9. Brown EC, Buchan DS, Baker JS, Wyatt FB, Bocalini DS, Kilgore L. A Systematised Review of Primary School Whole Class Child Obesity Interventions: Effectiveness, Characteristics, and Strategies. Biomed Res Int. 2016;2016.

10. Mei H, Xiong Y, Xie S, Guo S, Li Y, Guo B, et al. The impact of long-term school-based physical activity interventions on body mass index of primary school children - A meta-analysis of randomized controlled trials. BMC Public Health. 2016 Mar 1;16(1).

11. Oosterhoff M, Joore M, Ferreira I. The effects of school-based lifestyle interventions on body mass index and blood pressure: a multivariate multilevel meta-analysis of randomized controlled trials. Obesity Reviews. 2016 Nov 1;17(11):1131–53.

12. Bleich SN, Vercammen KA, Zatz LY, Frelier JM, Ebbeling CB, Peeters A. Interventions to prevent global childhood overweight and obesity: a systematic review. Lancet Diabetes Endocrinol. 2018 Apr 1;6(4):332–46.

13. Verjans-Janssen SRB, Van De Kolk I, Van Kann DHH, Kremers SPJ, Gerards SMPL. Effectiveness of school-based physical activity and nutrition interventions with direct parental involvement on children's BMI and energy balance-related behaviors – A systematic review. PLoS One. 2018 Sep 1;13(9):e0204560.

14. Wang Y, Cai L, Wu Y, Wilson RF, Weston C, Fawole O, et al. What childhood obesity prevention programmes work? A systematic review and meta-analysis. Obesity Reviews. 2015 Jul 1;16(7):547–65.

15. Liu Z, Xu HM, Wen LM, Peng YZ, Lin LZ, Zhou S, et al. A systematic review and meta-analysis of the overall effects of school-based obesity prevention interventions and effect differences by intervention components. Int J Behav Nutr Phys Act. 2019 Oct 29;16(1).

16. Brown T, Moore TH, Hooper L, Gao Y, Zayegh A, Ijaz S, et al. Interventions for preventing obesity in children. Cochrane Database of Systematic Reviews. 2019 Jul 23;2019(7).

17. Bagnall AM, Radley D, Jones R, Gately P, Nobles J, Van Dijk M, et al. Whole systems approaches to obesity and other complex public health challenges: A systematic review. BMC Public Health. 2019 Jan 3;19(1):1–14.

18. Waterlander WE, Singh A, Altenburg T, Dijkstra C, Luna Pinzon A, Anselma M, et al. Understanding obesity-related behaviors in youth from a systems dynamics perspective: The use of causal loop diagrams. Obesity Reviews. 2021 Jul 1;22(7):e13185.

19. Smith JD, Fu E, Kobayashi MA. Prevention and Management of Childhood Obesity and its Psychological and Health Comorbidities. Annu Rev Clin Psychol. 2020 May 5;16:351.

20. Amini M, Djazayery A, Majdzadeh R, Taghdisi MH, Jazayeri S. Effect of School-based Interventions to Control Childhood Obesity: A Review of Reviews. Int J Prev Med. 2015 Aug 3;6(AUGUST).

21. Bahia L, Schaan CW, Sparrenberger K, Abreu G de A, Barufaldi LA, Coutinho W, et al. Overview of meta-analysis on prevention and treatment of childhood obesity. J Pediatr (Rio J). 2019 Jul 1;95(4):385–400.

22. Shirley K, Rutfield R, Hall N, Fedor N, McCaughey VK, Zajac K. Combinations of obesity prevention strategies in US elementary schools: a critical review. J Prim Prev. 2015 Jan 1;36(1):1–20.

23. Waters E, de Silva-Sanigorski A, Burford BJ, Brown T, Campbell KJ, Gao Y, et al. Interventions for preventing obesity in children. Cochrane Database Syst Rev. 2011 Dec 7;2011(12).

24. Khambalia AZ, Dickinson S, Hardy LL, Gill T, Baur LA. A synthesis of existing systematic reviews and meta-analyses of school-based behavioural interventions for controlling and preventing obesity. Obes Rev. 2012 Mar;13(3):214–33.

25. Ickes MJ, McMullen J, Haider T, Sharma M. Global school-based childhood obesity interventions: a review. Int J Environ Res Public Health. 2014 Aug 28;11(9):8940–61.

26. Aceves-Martins M, Llauradó E, Tarro L, Moreno-García CF, Escobar TGT, Solà R, et al. Effectiveness of social marketing strategies to reduce youth obesity in European school-based interventions: a systematic review and meta-analysis. Nutr Rev. 2016 May 1;74(5):337–51.

27. Sobol-Goldberg S, Rabinowitz J, Gross R. School-based obesity prevention programs: A meta-analysis of randomized controlled trials. Obesity. 2013 Dec 1;21(12):2422–8.

28. Pablos A, Nebot V, Vañó-Vicent V, Ceca D, Elvira L. Effectiveness of a school-based program focusing on diet and health habits taught through physical exercise. Appl Physiol Nutr Metab. 2018;43(4):331–7.

29. Scherr RE, Linnell JD, Dharmar M, Beccarelli LM, Bergman JJ, Briggs M, et al. A Multicomponent, School-Based Intervention, the Shaping Healthy Choices Program, Improves Nutrition-Related Outcomes. J Nutr Educ Behav. 2017 May 1;49(5):368-379.e1.

30. Summerbell CD, Waters E, Edmunds L, Kelly SA, Brown T, Campbell KJ. Interventions for preventing obesity in children. Cochrane Database Syst Rev. 2005 Jul 20;(3).

31. Cerrato-Carretero P, Roncero-Martín R, Pedrera-Zamorano JD, López-Espuela F, Puerto-Parejo LM, Sánchez-Fernández A, et al. Long-Term Dietary and Physical Activity Interventions in the School Setting and Their Effects on BMI in Children Aged 6-12 Years: Meta-Analysis of Randomized Controlled Clinical Trials. Healthcare (Basel). 2021;9(4).

32. Economos CD, Hyatt RR, Goldberg JP, Must A, Naumova EN, Collins JJ, et al. A community intervention reduces BMI z-score in children: Shape Up Somerville first year results. Obesity (Silver Spring). 2007 May;15(5):1325–36.

33. Economos CD, Hyatt RR, Must A, Goldberg JP, Kuder J, Naumova EN, et al. Shape Up Somerville two-year results: A community-based environmental change intervention sustains weight reduction in children. Prev Med (Baltim). 2013 Oct;57(4):322–7.

34. Chomitz VR, McGowan RJ, Wendel JM, Williams SA, Cabral HJ, King SE, et al. Healthy Living Cambridge Kids: a community-based participatory effort to promote healthy weight and fitness. Obesity (Silver Spring). 2010 Feb;18 Suppl 1(SUPPL. 1).

35. Borys JM, Le Bodo Y, Jebb SA, Seidell JC, Summerbell C, Richard D, et al. EPODE approach for childhood obesity prevention: methods, progress and international development. Obes Rev. 2012 Apr;13(4):299–315.

36. De Silva-Sanigorski AM, Bell AC, Kremer P, Nichols M, Crellin M, Smith M, et al. Reducing obesity in early childhood: results from Romp & Chomp, an Australian community-wide intervention program. Am J Clin Nutr. 2010 Apr 1;91(4):831–40.

37. Sanigorski AM, Bell AC, Kremer PJ, Cuttler R, Swinburn BA. Reducing unhealthy weight gain in children through community capacity-building: results of a quasi-experimental intervention program, Be Active Eat Well. Int J Obes (Lond). 2008 Jul;32(7):1060–7.

38. Flodgren GM, Helleve A, Lobstein T, Rutter H, Klepp KI. Primary prevention of overweight and obesity in adolescents: An overview of systematic reviews. Obes Rev. 2020 Nov 1;21(11).

39. Reed H, Couturiaux D, Davis M, Edwards A, Janes E, Kim HS, et al. Co-production as an Emerging Methodology for Developing School-Based Health Interventions with Students Aged 11-16: Systematic Review of Intervention Types, Theories and Processes and Thematic Synthesis of Stakeholders' Experiences. Prev Sci. 2021 May 1;22(4):475–91.

40. Fødevarestyrelsen. Alt om kost - De officielle kostråd. www.altomkost.dk. 2021.

41. Nordic Council of Ministers. Nordic nutrition recommendations 2012. Integrating nutrition and physical activity. 5th ed. 2014.

42. Pedersen A, Christensen T, Matthiessen J, Knudsen V, Rosenlund-Sørensen M, Biltoft-Jensen A, et al. Danskernes kostvaner 2011-2013. Hovedresultater. Søborg: DTU Fødevareinstituttet; 2015.

43. Groth MV, Christensen LM, Knudsen VK, Sørensen MR, Fagt S, Ege M, et al. Sociale forskelle. Børns kostvaner, fysiske aktivitet og overvægt & voksnes kostvaner. 2013.

44. Andersen R, Biltoft-Jensen A, Christensen T, Andersen EW, Ege M, Thorsen A V., et al. Dietary effects of introducing school meals based on the New Nordic Diet - a randomised controlled trial in Danish children. The OPUS School Meal Study. Br J Nutr. 2014 Jun 14;111(11):1967–76.

45. Lundborg P, Rooth DO, Alex-Petersen J. Long-Term Effects of Childhood Nutrition: Evidence from a School Lunch Reform. Rev Econ Stud. 2022 Mar 6;89(2):876–908.

46. Rothausen BW, Matthiessen J, Andersen LF, Brockhoff PB, Tetens I. Dietary patterns on weekdays and weekend days in 4-14-year-old Danish children. Br J Nutr. 2013;109(9):1704–13.

47. Story M, Kaphingst KM, Robinson-O'Brien R, Glanz K. Creating healthy food and eating environments: policy and environmental approaches. Annu Rev Public Health. 2008;29:253–72.

48. Rasmussen M, Krølner R, Klepp KI, Lytle L, Brug J, Bere E, et al. Determinants of fruit and vegetable consumption among children and adolescents: a review of the literature. Part I: quantitative studies. Int J Behav Nutr Phys Act. 2006 Aug 11;3:22.

49. Bangsbo J, Krustrup P, Duda J, Hillman C, Andersen LB, Weiss M, et al. The Copenhagen Consensus Conference 2016: children, youth, and physical activity in schools and during leisure time. Br J Sports Med. 2016 Oct 1;50(19):1177–8.

50. Samdal O, Tynjälä J, Roberts C, Sallis JF, Villberg J, Wold B. Trends in vigorous physical activity and TV watching of adolescents from 1986 to 2002 in seven European Countries. Eur J Public Health. 2007 Sep;17(3):242–8.

51. Steene-Johannessen J, Anderssen SA, Kolle E, Hansen BH, Bratteteig M, Dalhaug EM, et al. Temporal trends in physical activity levels across more than a decade - a national physical activity surveillance system among Norwegian children and adolescents. Int J Behav Nutr Phys Act. 2021 Dec 1;18(1).

52. Robinson TN, Banda JA, Hale L, Lu AS, Fleming-Milici F, Calvert SL, et al. Screen Media Exposure and Obesity in Children and Adolescents. Pediatrics. 2017 Nov 1;140(Suppl 2):S97–101.

53. Morrissey B, Taveras E, Allender S, Strugnell C. Sleep and obesity among children: A systematic review of multiple sleep dimensions. Pediatr Obes. 2020 Apr 1;15(4).

54. Hale L, Guan S. Screen time and sleep among school-aged children and adolescents: a systematic literature review. Sleep Med Rev. 2015 Jun 1;21:50–8.

55. Pedersen J, Gillies M, Rasmussen B, Olesen G, Kristensen PL, Grøntved A. Self-administered electroencephalography-based sleep assessment: compliance and perceived feasibility in children and adults. Sleep Science and Practice 2021 5:1. 2021 Mar 3;5(1):1–12.

56. Rasmussen M, Kierkegaard L, Rosenwein SV, Holstein BE, Damsgaard MT, Due P. Skolebørnsundersøgelsen 2018. Helbred, trivsel og sundhedsadfærd blandt 11-, 13- og 15-årige skoleelever i Danmark. 2019.

57. Pedersen J, Rasmussen MGB, Sørensen SO, Mortensen SR, Olesen LG, Brønd JC, et al. Effects of Limiting Recreational Screen Media Use on Physical Activity and Sleep in Families With Children: A Cluster Randomized Clinical Trial. JAMA Pediatr. 2022;176(8).

58. Egan M, McGill E, Anderson de Cuevas R, Er V, Lock K, Popay J, et al. NIHR SPHR Guidance on Systems Approaches to Local Public Health Evaluation. Part 1: Introducing systems thinking. 2019.

59. Public Health England. Whole systems approach to obesity: A guide to support local approaches. 2019.

60. Moulton LH. Covariate-based constrained randomization of group-randomized trials. Clinical Trials. 2004 Jun 3;1(3):297–305.

61. Lloyd J, Creanor S, Logan S, Green C, Dean SG, Hillsdon M, et al. Effectiveness of the Healthy Lifestyles Programme (HeLP) to prevent obesity in UK primary-school children: a cluster randomised controlled trial. Lancet Child Adolesc Health. 2018 Jan;2(1):35–45.

62. Adab P, Pallan MJ, Lancashire ER, Hemming K, Frew E, Barrett T, et al. Effectiveness of a childhood obesity prevention programme delivered through schools, targeting 6 and 7 year olds: cluster randomised controlled trial (WAVES study). BMJ. 2018 Feb 7;k211.

63. Damsgaard CT, Dalskov SM, Laursen RP, Ritz C, Hjorth MF, Lauritzen L, et al. Provision of healthy school meals does not affect the metabolic syndrome score in 8–11-year-old children, but reduces cardiometabolic risk markers despite increasing waist circumference. British Journal of Nutrition. 2014 Dec 14;112(11):1826–36.

64. Damsgaard CT, Dalskov SM, Petersen RA, Sørensen LB, Mølgaard C, Biltoft-Jensen A, et al. Design of the OPUS School Meal Study: a randomised controlled trial assessing the

impact of serving school meals based on the New Nordic Diet. Scand J Public Health. 2012 Dec;40(8):693–703.

65. Pedersen NH, Koch S, Larsen KT, Kristensen PL, Troelsen J, Møller NC, et al. Protocol for evaluating the impact of a national school policy on physical activity levels in Danish children and adolescents: the PHASAR study - a natural experiment. BMC Public Health. 2018 Dec 8;18(1):1245.

66. Leyrat C, Morgan KE, Leurent B, Kahan BC. Cluster randomized trials with a small number of clusters: which analyses should be used? Int J Epidemiol. 2018 Feb 1;47(1):321–31.

67. Hudda MT, Aarestrup J, Owen CG, Cook DG, Sørensen TIA, Rudnicka AR, et al. Association of Childhood Fat Mass and Weight With Adult-Onset Type 2 Diabetes in Denmark. JAMA Netw Open. 2021 Apr 30;4(4):e218524.

68. Danmarks Statistik (DST) og Danmarks Tekniske Universitet F (DTU). Ulighed i børneovervægt i Danmark. 2021.

69. Danmarks Statistik (DST) og Danmarks Tekniske Universitet F (DTU). Ulighed i børneovervægt i Danmark. 2021.

70. Danmarks Statistik (DST) og Danmarks Tekniske Universitet F (DTU). Ulighed i børneovervægt i Danmark. 2021.

71. Danmarks Statistik (DST) og Danmarks Tekniske Universitet F (DTU). Ulighed i børneovervægt i Danmark. 2021.

72. Danmarks Statistik (DST) og Danmarks Tekniske Universitet F (DTU). Ulighed i børneovervægt i Danmark. 2021.

73. Bruun Meldgaard J, Bjerregaard LG, Heitmann BL, Due P, Høy TV, Kierkegaard L, et al. Forebyggelse af overvægt blandt børn og unge. . København; 2021.

74. Campbell M, Fitzpatrick R, Haines A, Kinmonth AL, Sandercock P, Spiegelhalter D, et al. Framework for design and evaluation of complex interventions to improve health. BMJ. 2000 Sep 16;321(7262):694–6.

75. Skivington K, Matthews L, Simpson SA, Craig P, Baird J, Blazeby JM, et al. Framework for the development and evaluation of complex interventions: gap analysis, workshop and consultation-informed update. Health Technol Assess. 2021;25(57):i–132.

76. Bloch P, Toft U, Reinbach HC, Clausen LT, Mikkelsen BE, Poulsen K, et al. Revitalizing the setting approach - supersettings for sustainable impact in community health promotion. Int J Behav Nutr Phys Act. 2014 Sep 14;11(1).

77. Hayward J, Morton S, Johnstone M, Creighton D, Allender S. Tools and analytic techniques to synthesise community knowledge in CBPR using computer-mediated participatory system modelling. npj Digital Medicine 2020 3:1. 2020 Feb 19;3(1):1–6.

78. Allender S, Orellana L, Crooks N, Bolton KA, Fraser P, Brown AD, et al. Four-Year Behavioral, Health-Related Quality of Life, and BMI Outcomes from a Cluster Randomized Whole of Systems Trial of Prevention Strategies for Childhood Obesity. Obesity (Silver Spring). 2021 Jun 1;29(6):1022–35.

79. Allender S, Owen B, Kuhlberg J, Lowe J, Nagorcka-Smith P, Whelan J, et al. A Community Based Systems Diagram of Obesity Causes. PLoS One. 2015 Jul 8;10(7).

80. Larsen MN, Nielsen CM, Helge EW, Madsen M, Manniche V, Hansen L, et al. Positive effects on bone mineralisation and muscular fitness after 10 months of intense school-based physical training for children aged 8-10 years: the FIT FIRST randomised controlled trial. Br J Sports Med. 2018 Feb 1;52(4):254–60.

81. Nielsen G, Pfister G, Andersen LB. Gender differences in the daily physical activities of Danish school children: http://dx.doi.org/101177/1356336X11402267. 2011 Jul 22;17(1):69–90.

82. Nielsen G, Bugge A, Hermansen B, Svensson J, Andersen LB. School playground facilities as a determinant of children's daily activity: a cross-sectional study of Danish primary school children. J Phys Act Health. 2012;9(1):104–14.

83. Toftager M, Pawlowski CS, Andersen HB, Christiansen LB, Schipperijn J, Troelsen J. Det aktive frikvarter - mere bevægelse i skolegården. 2019.

84. Belton S, McCarren A, McGrane B, Powell D, Issartel J. The Youth-Physical Activity Towards Health (Y-PATH) intervention: Results of a 24 month cluster randomised controlled trial. PLoS One. 2019 Sep 1;14(9).

85. Kok G, Gottlieb NH, Commers M, Smerecnik C. The ecological approach in health promotion programs: a decade later. Am J Health Promot. 2008;22(6):437–42.

86. Sallis JF, Owen N, Fisher EB. Ecological models of health behavior. In: Glanz K, Rimer BK, Viswanath K, editors. Health behavior and health education: Theory, research, and practice . Jossey-Bass; 2008. p. 465–85.

87. Hyseni L, Atkinson M, Bromley H, Orton L, Lloyd-Williams F, McGill R, et al. The effects of policy actions to improve population dietary patterns and prevent diet-related non-communicable diseases: scoping review. Eur J Clin Nutr. 2017 Jun 1;71(6):694–711.

88. Hollands GJ, Carter P, Anwer S, King SE, Jebb SA, Ogilvie D, et al. Altering the availability or proximity of food, alcohol, and tobacco products to change their selection and consumption. Cochrane Database Syst Rev. 2019 Sep 4;2019(9).

89. Burgoine T, Sarkar C, Webster CJ, Monsivais P. Examining the interaction of fast-food outlet exposure and income on diet and obesity: evidence from 51,361 UK Biobank participants. Int J Behav Nutr Phys Act. 2018 Jul 24;15(1).

90. Bernsdorf KA, Lau CJ, Andreasen AH, Toft U, Lykke M, Glümer C. Accessibility of fast food outlets is associated with fast food intake. A study in the Capital Region of Denmark. Health Place. 2017 Nov 1;48:102–10.

91. Glanz K, Bader MDM, Iyer S. Retail grocery store marketing strategies and obesity: an integrative review. Am J Prev Med. 2012;42(5):503–12.

92. Papas MA, Alberg AJ, Ewing R, Helzlsouer KJ, Gary TL, Klassen AC. The built environment and obesity. Epidemiol Rev. 2007 May;29(1):129–43.

93. Christensen U, Krølner R, Nilsson CJ, Lyngbye PW, Hougaard CO, Nygaard E, et al. Addressing social inequality in aging by the Danish occupational social class measurement. J Aging Health. 2014 Feb;26(1):106–27.

94. Ravens-Sieberer U, Auquier P, Erhart M, Gosch A, Rajmil L, Bruil J, et al. The KIDSCREEN-27 quality of life measure for children and adolescents: psychometric results from a cross-cultural survey in 13 European countries. Qual Life Res. 2007 Oct;16(8):1347–56.

95. Klakk H, Wester CT, Olesen LG, Rasmussen MG, Kristensen PL, Pedersen J, et al. The development of a questionnaire to assess leisure time screen-based media use and its proximal correlates in children (SCREENS-Q). BMC Public Health. 2020 May 12;20(1):1–12.

96. Owens JA, Spirito A, McGuinn M. The Children's Sleep Habits Questionnaire (CSHQ): Psychometric properties of a survey instrument for school-aged children. Sleep. 2000 Dec 15;23(8):1043–51.

97. Skotte J, Korshøj M, Kristiansen J, Hanisch C, Holtermann A. Detection of physical activity types using triaxial accelerometers. J Phys Act Health. 2014 Jan;11(1):76–84.

98. Brønd JC, Grøntved A, Andersen LB, Arvidsson D, Olesen LG. Simple Method for the Objective Activity Type Assessment with Preschoolers, Children and Adolescents. Children (Basel). 2020 Jul 1;7(7).

99. Bendiksen M, Ahler T, Clausen H, Wedderkopp N, Krustrup P. The use of Yo-Yo intermittent recovery level 1 and Andersen testing for fitness and maximal heart rate assessments of 6- to 10-year-old school children. J Strength Cond Res. 2013 Jun;27(6):1583–90.

100. Geertsen SS, Thomas R, Larsen MN, Dahn IM, Andersen JN, Krause-Jensen M, et al. Motor Skills and Exercise Capacity Are Associated with Objective Measures of Cognitive Functions and Academic Performance in Preadolescent Children. PLoS One. 2016 Aug 1;11(8).

101. Cromer JA, Schembri AJ, Harel BT, Maruff P. The nature and rate of cognitive maturation from late childhood to adulthood. Front Psychol. 2015 May 27;6:704.

102. Sorensen LB, Dyssegaard CB, Damsgaard CT, Petersen RA, Dalskov SM, Hjorth MF, et al. The effects of Nordic school meals on concentration and school performance in 8- to 11-year-old children in the OPUS School Meal Study: a cluster-randomised, controlled, cross-over trial. Br J Nutr. 2015 Apr 28;113(8):1280–91.

103. Haerens L, Aelterman N, Vansteenkiste M, Soenens B, Van Petegem S. Do perceived autonomy-supportive and controlling teaching relate to physical education students' motivational experiences through unique pathways? Distinguishing between the bright and dark side of motivation. Psychol Sport Exerc. 2015 Mar 1;16(P3):26–36.

104. McAuley ED, Duncan T, Tammen V V. Psychometric Properties of the Intrinsic Motivation Inventory in a Competitive Sport Setting: A Confirmatory Factor Analysis. https://doi.org/101080/02701367198910607413. 2013;60(1):48–58.

105. Markland D, Hardy L. On the Factorial and Construct Validity of the Intrinsic Motivation Inventory: Conceptual and Operational Concerns. http://dx.doi.org/101080/02701367199710608863. 2013;68(1):20–32.

106. Elsborg P, Melby PS, Kurtzhals M, Tremblay MS, Nielsen G, Bentsen P. Translation and validation of the Canadian assessment of physical literacy-2 in a Danish sample. BMC Public Health. 2021 Dec 1;21(1).

107. Maitland N, Wardle K, Whelan J, Jalaludin B, Creighton D, Johnstone M, et al. Tracking implementation within a community-led whole of system approach to address childhood overweight and obesity in south west Sydney, Australia. BMC Public Health. 2021 Jun 26;21(1):1233.

108. Victorian Health Promotion Foundation. The partnerships analysis tool, Victorian Health Promotion Foundation. 2011.

109. Indig D, Grunseit A, Greig A, Lilley H, Bauman A. Development of a tool for the evaluation of obesity prevention partnerships. Health Promot J Austr. 2019 Jan 1;30(1):18–27.

110. Bell ML, Rabe BA. The mixed model for repeated measures for cluster randomized trials: a simulation study investigating bias and type I error with missing continuous data. Trials. 2020 Dec 7;21(1):148.

111. Twisk J, de Boer M, de Vente W, Heymans M. Multiple imputation of missing values was not necessary before performing a longitudinal mixed-model analysis. J Clin Epidemiol. 2013 Sep;66(9):1022–8.

11 List of Appendices (all in Danish)

Appendix 1: Protokol resumé

Appendix 2A: Spørgeskema om fødevareallergi, allergi over for plaster, kronisk sygdom og andre hensyn

Appendix 2B: Spørgeskema om allergi over for plaster, kronisk sygdom og andre hensyn (kontrolskoler)

Appendix 3: Drop-out skema

Appendix 4A og 4B: Invitationsbrev til interventionsskoler og kontrolskoler

Appendix 5A og 5B: Deltagerinformation til interventionsskoler og kontrolskoler

Appendix 6A og 6B: Samtykkeerklæring (Komitéens Standardblanketter S5 og S6)

Appendix 7: Forældrefuldmagt

Appendix 8A og 8B: Pjece om informationsmøde til interventionsskoler og kontrolskoler

Appendix 9: Plakat om informationsmøde

Appendix 10: Spørgeskema om familiebaggrund, idræt og solvaner

Appendix 11: Spørgeskema om pubertetsudvikling til forældre med drenge

Appendix 12: Spørgeskema om pubertetsudvikling til forældre med piger

Appendix 13: Spørgeskema om barnets kost

Appendix 14: Spørgeskemaet KIDSCREEN 27

Appendix 15: SCREENS modificeret spørgeskema

Appendix 16: Børns søvnvaner spørgeskemaet

Appendix 17: Uønskede hændelser og samtidig medicin

Appendix 18: Spørgeskema om dækning af basale psykologiske behov og IM under interventionsaktiviteter

Appendix 19: Spørgeskema til børn om physical literacy

Appendix 20: Spørgeskema til børn om deltagelse i fritidsidræt

Appendix 21: Spørgeskema om madkompetence og måltidskultur

Appendix 22: Diplom

Appendix 23: Procesevalueringsspørgeskema for børn

Appendix 24: Smileyskala til børneevaluering af interventionskomponenter

Appendix 25: Spørgeskema til forældre om deres indkøbsvaner

Appendix 26: Forældrerapporteret trivsel

Appendix 27A, B, C, D: Videoer om BODPOD, bevægelsesmåler, blodtryk og blodprøve
