# Supplementary material for: Generation Healthy Kids: Protocol for a cluster-randomized controlled trial of a multi-component and multi-setting intervention to promote healthy weight and wellbeing in 6–11-year-old children in Denmark
Source: PLoS One. 2024 Dec 5;19(12):e0308142. doi: 10.1371/journal.pone.0308142 (PMC11620443; doi:10.1371/journal.pone.0308142)
Supplement: Attachment — Submitted filename: Response to reviewer and editor comments_PONE-D-24-05389.docx [file pone.0308142.s005.docx]

**Response to editor comments:**

**Please note:**

**All page- and line numbers in the following refer to the “tracked changes”-version of the revised manuscript.**

*Dear authors,*

*Thank you for submitting your manuscript entitled “Generation Healthy Kids: A Multi-Setting, Multi-Component Intervention to Promote Healthy Weight Development in Danish Children Aged 6–11 Years” to PLOS ONE.*

*Your study protocol is well-written and outlines an impressive and comprehensive approach to addressing childhood obesity. The design of the GHK study, utilizing a cluster-randomized trial across multiple schools and incorporating diverse settings, is robust and promising.
The emphasis on capacity building and stakeholder involvement is particularly noteworthy, offering a novel strategy to promote child health and prevent obesity.*

Author response:

Thank you for your positive comments.

*Reviewer 1 has provided some valuable comments and suggestions that can further strengthen your manuscript. I recommend that you carefully consider and incorporate these comments in your revision.*

Author response:

All comments from Reviewer 1 are addressed below and in the revised manuscript.

*I only have a few minor comments (all are mentioned by reviewer 1 as well):*

*L389 Consent: Do you obtain consent from one or both if there is shared custody?*

Author response:

If there is shared custody, both custody holders must provide informed consent. Alternatively, one custody holder can grant a written power of attorney to the other to consent on their behalf. This is now explained on p. 20, lines 439–440.

*L441: will all children get the free lunch irrespective of they participate in the study or not?*

Author response:

Thank you for encouraging us to clarify this important point. To ensure equal treatment, intervention components implemented in schools and afterschool clubs will be offered to all children, irrespective of whether they participate in the study’s biomedical measurement schedule. Thus, all children will be offered the free lunch and will be able to participate in the extra physical activity during school hours. Likewise, all families will be invited to family events organized as part of the study, regardless of their child’s enrolment in the measurement protocol.

This was previously described in the Ethics sections (p. 47, lines 966–968), and we have now made it more clear in the section describing the overall intervention structure (p. 22, lines 481–486).

*L640 data: is it voluntary for the children? Can they decide to not do the tests on the assessment days if there is consent from parents?*

Author response:

Yes, participation in the measurement schedule is completely voluntary for the children. On the measurement days, if a child does not want to participate, or if the child expresses discomfort or uneasiness with the procedure, the measurement will not be performed, even though there is parental consent.

This is clarified in the revised manuscript on p. 33, lines 744–746, and p. 46, lines 958–959.

*Thank you for your valuable contribution to the field. I look forward to receiving your revised manuscript.*

Author response:

Thank you for taking the time to consider our manuscript and for your constructive inputs.

**Response to reviewer comments:**

**Reviewer #1:**

*Thank you for the opportunity to review this paper, which addresses the crucial topic of childhood obesity. The authors have outlined a protocol for a comprehensive intervention targeting children aged 6-11 and its implementation from the children's perspective.*

*Overall, the paper is well-written, and the study and intervention are clearly described. However, it would benefit from adhering to the CONSORT guidelines to enhance its rigor. While the authors plan to use CONSORT when publishing the results, the protocol itself would also benefit from following these guidelines.*

Author response:

Thank you for your constructive inputs. We have chosen to follow the SPIRIT Guidelines for Reporting of Protocols for Clinical Trials as required by PLOS One submission guidelines. The SPIRIT guidelines are the standard reporting guidelines for reporting of clinical trial protocols, whereas the CONSORT guidelines will be used for reporting the trial results. We have uploaded a completed SPIRIT checklist as Additional File 1.

*The intervention description is generally clear, but could be improved by using a standardized framework such as the TIDieR tool. I have some specific comments and suggestions for improving the manuscript.*

Author response:

Thank you for your comment. We agree that the TIDieR tool is a useful checklist for ensuring comprehensive description of intervention elements, and we have strived to include all TIDieR elements in our description of the intervention (pp. 21–32, lines 450–715).

In the revised manuscript, we have included further details on certain intervention elements as requested by TIDieR guidelines, including:

- A new Supplementary Table 3 has been added which provides a detailed overview of all intervention components, including their timing, duration, and frequency, as well as the provider responsible for delivering the intervention component.
- We have added more elaborated descriptions of modes of delivery, details of intervention procedures, details on timing and frequency, and tailoring of the intervention for certain intervention components (p. 24, lines 528–530; p. 25, line 547–548; p. 25, lines 556–562; p. 26, lines 578–580).

We have also uploaded a completed TIDieR checklist as a new Additional File 4 to demonstrate our adherence to these guidelines (Additional File 4).

***Abstract:***

*The sample size should be mentioned in more detail, including a justification (power analysis) for the targeted sample size. The abstract also lacks a detailed description of the outcomes, measurement time points, and focuses more on the intervention itself. Additionally, the timing of the study implementation is missing.*

Author response:

Thank you for your comment. We have inserted a description of the timing of study implementation and the timing of measurements in the abstract (p. 2, lines 59 and 68). The primary outcome is already described (p. 2–3, lines 68–70). We have now also noted that we have numerous secondary outcomes and other prespecified outcomes related to child health and wellbeing (p. 3, lines 70–71).

We found that explaining the power calculation was too lengthy to be included in the abstract, and this is not recommended in CONSORT guidelines for abstracts (1). The sample size calculation is explained in detail in the Methods section of the manuscript (p. 13, lines 302–321).

*Background:*

*The flow is good, and the intervention and its goals, as well as the state of the art, are well-described. Starting at line 145, the intervention description begins. Several approaches form the basis for the intervention, such as the population-based primary prevention approach (line 147) and the whole-system approach (line 190), but there is no information about what these approaches entail or the references they are based on. Readers would benefit from a few sentences elaborating on these approaches. Since the intervention aims for behavioral changes, it would be helpful to describe the theoretical framework underpinning the intervention.*

Author response:

Thank you for your comment. The whole-systems approach to obesity prevention is thoroughly described on p. 5–6, lines 126–139 with associated references. We have now additionally inserted references about the whole-systems approach on p. 8, line 201, as requested by the reviewer. We have also inserted a reference to a description of the population-based primary prevention approach in public health (2) (p. 6, line 152). Finally, we have added that the intervention is based on an ecological approach to health behavior and health promotion (p. 6, lines 152–155).

*Terms like "lower" and "most" in lines 160 and 174 should be accompanied by precise numbers to avoid vagueness, as seen with the "10%" mentioned in line 185.*

Author response:

We agree. We have now inserted more precise information on the proportions of Danish children failing to meet national and international recommendations for dietary intake and physical activity (p. 7, lines 169–171; p. 7–8, lines 182–183).

*The sentence in lines 188-190 is complex and needs clarification, particularly about who the stakeholders are.*

Author response:

We agree. This sentence is meant as a summary of the overall design and content of the intervention, which is then described in further detail in the Methods section, including the involvement of local stakeholders. We have now clarified this on p. 8, lines 201–202). On p. 29–30, lines 658–662 we describe in detail the stakeholders included in the local community intervention.

*Additionally, the paper lacks a cohesive description of the intervention's development, as it is scattered across different sections, making it difficult to follow. Although the pilot and feasibility study (line 199) will be reported in a separate paper, it still needs further explanation regarding sample sizes, what was measured, and when and where these measurements took place.*

Author response:

We agree. We have now inserted a separate section which describes the development of the intervention (p. 9–10, lines 220–248). We have also inserted more details on the pilot study, including the purpose, timing, setting, sample size, and data collection in the pilot (p. 10, lines 240–249).

*The paragraph starting at line 209 includes a list-like text, and some descriptions would be better placed in the methods section, especially the list of outcomes.*

Author response:

Thank you for your comment. We have now changed the text to avoid a list-like format of the Aims section (p. 11, lines 257–267).

*From line 230, if the authors are following a framework for implementation science, it should be mentioned to justify the measures taken to evaluate the intervention's implementation.*

Author response:

Thank you for your comment. The process evaluation is guided by the UK Medical Research Council’s framework for developing and evaluating complex interventions and guidance for process evaluation (3,4). Key concepts from the Consolidated Framework of Implementation Research (5) and the RE-AIM framework (6) will be used to evaluate the potential for sustainability and scale-up of the intervention. This is now added on p. 37, lines 837–841.

**Design and Methods:**

*As previously mentioned, the methods section would benefit from being structured according to CONSORT guidelines.*

Author response:

Thank you for your comment. We have chosen to structure the Methods section according to the SPIRIT guidelines, as required by the author guidelines of PLOS One (please see Additional File 1 for the completed SPIRIT statement, showing full compliance with all points in the SPIRIT checklist).

*In line 250, please specify the eligibility criteria for the schools.*

Author response:

The eligibility criteria and invitation process for schools are described in detail on p. 14–15, lines 341–353 and are shown in Figure 3.

*In line 319, clarify the requirements for the schools.*

Author response:

We have now included a full list of the requirements for schools to participate in the project (see p. 15, line 363 and Additional File 2, Supplementary Table 2).

*Regarding line 389, was there a reason for not seeking consent from the children? While parental consent is necessary, children are capable of providing their own informed consent. This issue should be addressed and elaborated upon in the ethics section, including how researchers ensure the willingness and voluntary participation of the children.*

Author response:

Thank you, we agree that this is a very important point. According to Danish law (Law on Health Research Ethics §4, §4A and §5), children under the age of 15 cannot legally provide informed consent for participation in a biomedical research study. However, researchers are obligated to ensure that children are thoroughly informed about the study, and the child’s perspectives must be considered to ensure that participation is completely voluntary for the child.

We will ensure this in the following ways:

- Prior to provision of informed consent, the study will be carefully explained to all children in a language suitable for their age and comprehension (in the classroom, at information meetings, and/or at bilateral meetings with parents and child).
- Furthermore, parents will be asked to discuss the study with their child and take the child’s perspectives into account before they decide whether to provide informed consent for their child.
- Both parents and children will be informed that participation is completely voluntary, and that consent can be withdrawn at any time during the study.
- On measurement days, if a child declines participation or exhibits any signs of discomfort or unease with the procedures, the measurements will not be performed, regardless of the custody holders’ provision of informed consent. Great care will be taken to ensure that all measurements are performed in a pleasant and safe atmosphere for the children.

In the revised manuscript, we have clarified these points in greater detail, please see revised manuscript p. 19, line 428; p. 20, lines 434–441; p. 33, lines 745–747; p. 45, lines 947–949, p. 45–46, lines 956–960; and p. 46, lines 965–967.

*In lines 407 and 409, the authors mention core elements and co-creation elements. However, in the detailed intervention description that follows, the distinction between these elements is not consistently mentioned. A detailed table outlining these elements, including their frequency, timing (e.g., whether they last the entire two years and start from the beginning), target groups, and other critical aspects of their implementation, would help readers better understand the complex intervention. If not presented in a table, the text should include these important details to demonstrate the rigor of the intervention.*

Author response:

Thank you, this is a good point. We apologize that the distinction between “core” and “co-created” elements was not sufficiently clear. We have now provided further clarification on how we defined “core” and “co-created” elements on p. 21, lines 462–464, and we have slightly updated Figure 4 to make the difference clearer.

As recommended by the reviewer, we have furthermore inserted a Table which provides a detailed overview of all intervention components, including their frequency and timing, and who is responsible for delivering the intervention. This table clearly shows which parts of the intervention are considered “core” and “co-created” components, respectively (Additional File 2, Supplementary Table 3).

*Line 419 discusses the development of the intervention and would be better placed in the section detailing the development process, prior to the methods section. A process graph illustrating all phases of the development process would also be beneficial.*

Author response:

Thank you, we agree with this point. To clarify the intervention development process, we have now inserted a separate section prior to the Methods section which describes the development of the intervention (p. 9–10, lines 225–239). As suggested, the description of how we involved national stakeholders in the process has been moved to this section (page 9–10, lines 225–239).

*Additionally, it is important to mention if children, parents, or teachers were included in the development process, as this participatory approach is crucial in designing and developing health interventions.*

School staff, school management and parents were involved in the evaluation of the pilot- and feasibility study, and thereby they provided valuable inputs to the intervention development process. Inputs from children were included indirectly through comprehensive research staff observations of activities conducted in schools and afterschool clubs during the pilot. This is now mentioned in the manuscript (p. 10, lines 247–248), and it will be described in detail in a separate publication *(Lund L, Brautsch LAS, Hoeeg D et al., Feasibility and acceptability of school-based intervention components to promote healthy weight and wellbeing among 6–11-year-olds in Denmark: mixed methods findings from the Generation Healthy Kids feasibility study. Submitted June 2024.)*.

In the main trial, children will be involved in the co-creation of local action ideas implemented by the Local Community Partnership Groups as described in the manuscript on p. 30–31, lines 686–697.

*From line 440, it is unclear what happens to children who do not participate in the study. Will they still receive the intervention? It is important to mention that non-participating children will not be treated unequally.*

Author response:

All children and families in the included classes will be offered the intervention activities, including free lunch, physical activity in the school curriculum, classroom exercises and invitations for family events, irrespective of whether the child is enrolled in the biomedical measurement schedule. This is to ensure that no children are treated unequally or feel left out in the classroom.

We have now clarified this on p. 22, lines 483–488, and it is also mentioned in the Ethics section (p. 46, lines 968–970).

*The section from lines 482-486 is brief and needs more elaboration on the content of this element.*

Author response:

We agree, and we have now described the content of the classroom exercises more elaborately (p. 25, lines 558–564).

*For example, lines 534 and 553 describe possible differences between interventions in different contexts. How will the authors ensure that the results are not affected by these differences if the intervention is not fully standardized?*

Author response:

The GHK intervention is based on whole systems thinking (7,8) and the Supersetting approach (9) and intervention development was inspired by the UK Medical Research Council’s guidelines for complex interventions (3). These approaches stress that complex interventions must be sensitive to the local context in which the intervention is embedded. Thus, in accordance with the theoretical foundation of GHK, the intervention must be adapted and tailored to local needs, structures, opportunities, and barriers in each of the intervention schools and communities (9).

As explained in the manuscript, some GHK intervention components (referred to as “core” components) were predefined based on preexisting evidence and prior experience in our research group. These components will be implemented in a uniform and standardized manner at all intervention schools, and the core components are mandatory for the research group and school staff to deliver at all 12 schools *(p. 10, lines 241–243; p. 21, lines 463–468; Supplementary Table 3).*

Other intervention elements (referred to as “co-created” components) will be tailored to the local context in schools and local communities. The specific content and design of these elements will be decided through direct interaction and dialogue with local stakeholders, including the school management, staff, and local community partnership groups, by identifying what is relevant and realistic in their view, and by jointly planning and implementing activities with the local stakeholders (9) (p. 21, lines 468–472; Supplementary Table 3). These elements will thus not be standardized across the intervention areas.

This combination of predefined and co-created elements is a unique feature of Generation Healthy Kids, which to our knowledge has not previously been tested in a large intervention study of childhood obesity prevention (p. 47–48, lines 1004–1007).

In the process evaluation, we will thoroughly document the delivery of elements in each setting, including what has been delivered to whom, where and when, in order to understand the working mechanisms of the intervention in different contexts (p. 37, lines 836–849).

In the revised manuscript, the Supersetting and whole-systems approaches are described on p. 5–6, lines 126–139 and p. 8, lines 202–205. We have added a more elaborate description of the distinction between “core” and “co-created” intervention components on p. 21, lines 463–468; p. 48, lines 1004–1007; Figure 4; and Supplementary Table 3 (Additional File 2). Finally, the process evaluation, including how we will document the intervention components delivered, is described on p. 37, lines 836–849.

*From line 538, it is unclear when the children's visions are communicated to other stakeholders and what actions will follow.*

Author response:

Thank you for this valid comment. The children’s visions and ideas will be communicated to the school management and the local community partnership groups (LPCGs) in the middle of school year 2. The specific actions will depend on the ideas articulated by the children. Where feasible and relevant, the research team will facilitate and support the LPCGs in implementing the changes and ideas brought forward by the children.

This is now clarified on p. 31, lines 686–697 and in Supplementary Table 3 (Additional File 2). We have moved the paragraph on involvement of children to the section on “Interventions in the Local Community” to further highlight that the LPCGs will be responsible for implementing the children’s ideas.

*Overall, there are instances where the authors describe actions performed by the research group and refer to "us." Are "we" the same as "the research group"? This should be clarified.*

Author response:

Yes, “we” refers to “the research group”. We have opted to mainly use active language (e.g., “we invited public schools”) instead of passive language (e.g. “public schools were invited”), because we believe this improves clarity, and the active style is recommended by many medical journals, for example BMJ (<https://www.bmj.com/about-bmj/resources-authors/house-style>). Using the term “we” instead of “research group” provides brevity and clarity. We think this is standard practice in contemporary academic medical writing, and it has been used in study protocols recently published in PLOS One (10–12).

*From line 714, explain how the effects of the food environment intervention are calculated.*

Author response:

OK, we have now added more details on the sales data collected from supermarkets (p. 36–37, lines 821–833) and how the effects will be calculated (p. 44, lines 920–924).

*From line 722, provide more details about the field observations, focus groups, interviews, and surveys, including what is evaluated, the sample size, and the timing of these evaluations.*

Author response:

The planned process evaluation is very comprehensive, and it involves extensive data collection of both quantitative (questionnaires, logbooks) and qualitative (field observations, interviews, focus groups) data from several sources (school management, staff, parents, children, and local community members). To allow a sufficiently clear and detailed description of the process evaluation protocol, we consider it appropriate to publish a separate protocol paper on this. Therefore, we have only provided a summary in the present manuscript.

We have now stressed this more clearly in the beginning of the section “Process evaluation data” (p. 37, lines 842–844).

***Ethical Considerations:***

*As mentioned regarding the consent issue, the section also needs a more detailed reflection on the study's impact on children. The numerous measurements and questionnaires might be exhausting for the children, and this should be considered and addressed.*

Author response:

Thank you, we agree that the study’s potential impact on the children is very important, and we have carefully considered the potential effects of the comprehensive measurement schedule. In the revised manuscript, we have added more details on our considerations in the Ethics section, including:

- Measurement days are organized within the time frame of normal school days, and there is time for breaks to avoid exhausting the children.
- If a child exhibits discomfort or unease during the procedures, or if the child does not want to participate, the measurement is discontinued, even though there is parental consent.
- All communication to families and children occurs in a non-stigmatizing manner, focusing on wellbeing, general health, and sense of community, with no focus on weight or body size.
- It is a part of the study’s process evaluation to investigate how children experience the measurement days.

Please see revised manuscript p. 45–46, lines 957–963.

***Discussion:***

*In line 868, it is unclear how the co-creation elements were developed and implemented. More detailed information on the co-creation process is needed. This should include how stakeholders, including children, parents, teachers, and other relevant parties, were involved in the co-creation process, and the specific steps taken to ensure their meaningful participation.*

Author response:

Co-creation processes are performed at several levels in the study:

- The co-creation process with national stakeholders prior to intervention start is described on p. 9–10, lines 225–239.
- The co-creation process in local communities is described on p. 29–30, lines 648–684.
- The co-creation process involving children is described on p. 30, lines 686–697.

In the revised manuscript, we have provided more details on the co-creation processes on p. 21, line 468–472 and p. 24, lines 530–532.

*Thank you for the opportunity to review your paper. I wish you all the best in implementing your intervention. Your work on addressing childhood obesity is vital and commendable, and I look forward to seeing the results of this important study.*

Author response:

We thank you sincerely for taking the time to review our work and for your valuable comments which have helped us improve the manuscript.

**References**

1.	Hopewell S, Clarke M, Moher D, Wager E, Middleton P, Altman DG, et al. CONSORT for Reporting Randomized Controlled Trials in Journal and Conference Abstracts: Explanation and Elaboration. PLoS Med. 2008;5(1):48–56.

2.	McLaren L. In defense of a population-level approach to prevention: why public health matters today. Can J Pub Health. 2019;110(3):279–84.

3.	Skivington K, Matthews L, Simpson SA, Craig P, Baird J, Blazeby JM, et al. A new framework for developing and evaluating complex interventions: Update of Medical Research Council guidance. BMJ. 2021;374:n2061.

4.	Moore GF, Audrey S, Barker M, Bond L, Bonell C, Hardeman W, et al. Process evaluation of complex interventions: Medical Research Council guidance. BMJ. 2015;350:h1258.

5.	Damschroder LJ, Reardon CM, Widerquist MAO, Lowery J. The updated Consolidated Framework for Implementation Research based on user feedback. Implement Sci. 2022;17:75.

6.	Glasgow RE, Harden SM, Gaglio B, Rabin B, Smith ML, Porter GC, et al. RE-AIM planning and evaluation framework: Adapting to new science and practice with a 20-year review. Front Pub Health. 2019;7:64.

7.	Public Health England. Whole systems approach to obesity: A guide to support local approaches to promoting a healthy weight. London; 2019.

8.	Bagnall AM, Radley D, Jones R, Gately P, Nobles J, Van Dijk M, et al. Whole systems approaches to obesity and other complex public health challenges: A systematic review. BMC Public Health. 2019;19(1):8.

9.	Bloch P, Toft U, Reinbach HC, Clausen LT, Mikkelsen BE, Poulsen K, et al. Revitalizing the setting approach - supersettings for sustainable impact in community health promotion. Int J Behav Nutr Phy Act. 2014;11(1):118.

10.	Mkumbuzi NS, Massey A, Lubega SK, Sorowen B, Chisati EM. FIFA football nurse – A task sharing approach in sports and exercise medicine practice in grassroots women’s football in low- and middle- income settings. A study protocol for a cluster randomised controlled trial. PLoS One. 2023;18(9):e0278428.

11.	Willis EA, Burney R, Hales D, Ilugbusi LAO, Tate DF, Nezami B, et al. “My wellbeing-their wellbeing “– An eHealth intervention for managing obesity in early care and education: Protocol for the Go NAPSACC Cares cluster randomized control trial. PLoS One. 2023;18(7):e0286912.

12.	Wahi G, Kandasamy S, Bangdiwala SI, Baumann A, Crea-Arsenio M, Desai D, et al. Strengthening Community Roots: Anchoring Newcomers in Wellness and Sustainability (SCORE!): A protocol for the co-design and evaluation of a healthy active living program among a newcomer community in Canada. PLoS One. 2023;18(9):e0288851.
